# The Rab11-binding protein RELCH/KIAA1468 controls intracellular cholesterol distribution

**DOI:** 10.1083/jcb.201709123

**Published:** 2018-05-07

**Authors:** Tomoaki Sobajima, Shin-ichiro Yoshimura, Tomomi Maeda, Haruhiko Miyata, Eiji Miyoshi, Akihiro Harada

**Affiliations:** 1 Department of Cell Biology, Graduate School of Medicine, Osaka University, Osaka, Japan; 2 Department of Molecular Biochemistry and Clinical Investigation, Graduate School of Medicine, Osaka University, Osaka, Japan; 3 Department of Experimental Genome Research, Research Institute for Microbial Diseases, Osaka, Japan

## Abstract

Cholesterol, which is endocytosed to the late endosome (LE)/lysosome, is delivered to other organelles through vesicular and nonvesicular transport mechanisms. In this study, we discuss a novel mechanism of cholesterol transport from recycling endosomes (REs) to the trans-Golgi network (TGN) through RELCH/KIAA1468, which is newly identified in this study as a Rab11-GTP– and OSBP-binding protein. After treating cells with 25-hydroxycholesterol to induce OSBP relocation from the cytoplasm to the TGN, REs accumulated around the TGN area, but this accumulation was diminished in RELCH- or OSBP-depleted cells. Cholesterol content in the TGN was decreased in Rab11-, RELCH-, and OSBP-depleted cells and increased in the LE/lysosome. According to in vitro reconstitution experiments, RELCH tethers Rab11-bound RE-like and OSBP-bound TGN-like liposomes and promotes OSBP-dependent cholesterol transfer from RE-like to TGN-like liposomes. These data suggest that RELCH promotes nonvesicular cholesterol transport from REs to the TGN through membrane tethering.

## Abstract (precis)

Sobajima et al. identify the novel protein RELCH/KIAA1468 as a Rab11-binding protein and show that RELCH/KIAA1468 and Rab11 regulate OSBP-dependent nonvesicular cholesterol transport from recycling endosomes to the trans-Golgi network.

## Introduction

Most mammalian cells acquire cholesterol through the endocytosis of plasma lipoproteins such as low-density lipoprotein (LDL). After LDL is delivered to the lysosome, free cholesterol, which is derived from hydrolyzed cholesterol ester liberated from LDL, is transported from the lysosome to various subcellular membrane compartments (Ioannou, 2001; Ikonen, 2008).

Accumulating evidence suggests that intracellular cholesterol transport is mediated by the following two mechanisms: vesicular and nonvesicular transport. In vesicular transport, SNARE proteins, which mediate vesicle/membrane fusion, are involved in cholesterol delivery from the endosome to the trans-Golgi network (TGN; Urano et al., 2008). In nonvesicular transport, oxysterol binding protein–related proteins (ORPs) are potential key regulators.

Several ORPs are localized at membrane contact sites (MCSs) and mediate lipid transfer between organelle membranes (Olkkonen, 2015). In addition, the oxysterol-binding protein (OSBP)-related ligand binding domain (ORP-related domain [ORD]) of ORPs binds lipids such as oxysterol, ergosterol, cholesterol, phosphatidylinositol (PI), and phosphatidylserine (PS; Im et al., 2005; Maeda et al., 2013; Mesmin et al., 2013; Liu and Ridgway, 2014), suggesting that ORPs function as lipid sensors or lipid transfer proteins at MCSs. OSBP, which is a TGN-localized protein, is among the best characterized ORPs. OSBP transfers cholesterol from the ER to the TGN through the countertransfer of PI 4-phosphate (PI4P) at ER–Golgi MCSs (Mesmin et al., 2013).

The Rab GTPase family, which comprises 60 members in mammals, regulates various steps in intracellular protein transport such as vesicle/tubule generation, motility along the cytoskeleton, tethering, and fusion by recruiting specific binding proteins to the membrane (Stenmark, 2009). Several studies have suggested that certain Rab proteins, such as Rab8, Rab9, and Rab11, and their effector proteins are involved in intracellular cholesterol transport (Hölttä-Vuori et al., 2002; Narita et al., 2005; Kanerva et al., 2013).

Rab11 is a highly conserved eukaryotic protein (Rab11a and Rab11b are the two paralogs encoded by the human genome) localized to the recycling endosomes (REs). Rab11 has been implicated in the exocytic and endocytic recycling pathways to the plasma membrane (PM) and ciliary membrane biogenesis (Ullrich et al., 1996; Chen et al., 1998; Knödler et al., 2010). In a previous study, the reesterification of cellular cholesterol, which is catalyzed by ER-resident acyl-coenzyme A-cholesterol acyltransferase, was reduced in Rab11-overexpressing cells, indicating that Rab11 or RE function is involved in intracellular cholesterol transport (Hölttä-Vuori et al., 2002). However, the precise molecular role of Rab11 in cholesterol transport is poorly understood.

In this article, we present a novel role of Rab11 in cholesterol transfer from REs to the TGN; RELCH/KIAA1468, which is a newly identified Rab11 effector protein, tethers the RE and TGN membranes by binding TGN-localized OSBP and promotes OSBP-dependent nonvesicular cholesterol transport from REs to the TGN.

## Results

### RELCH/KIAA1468 is a novel Rab11-binding protein

**Figure 1.**
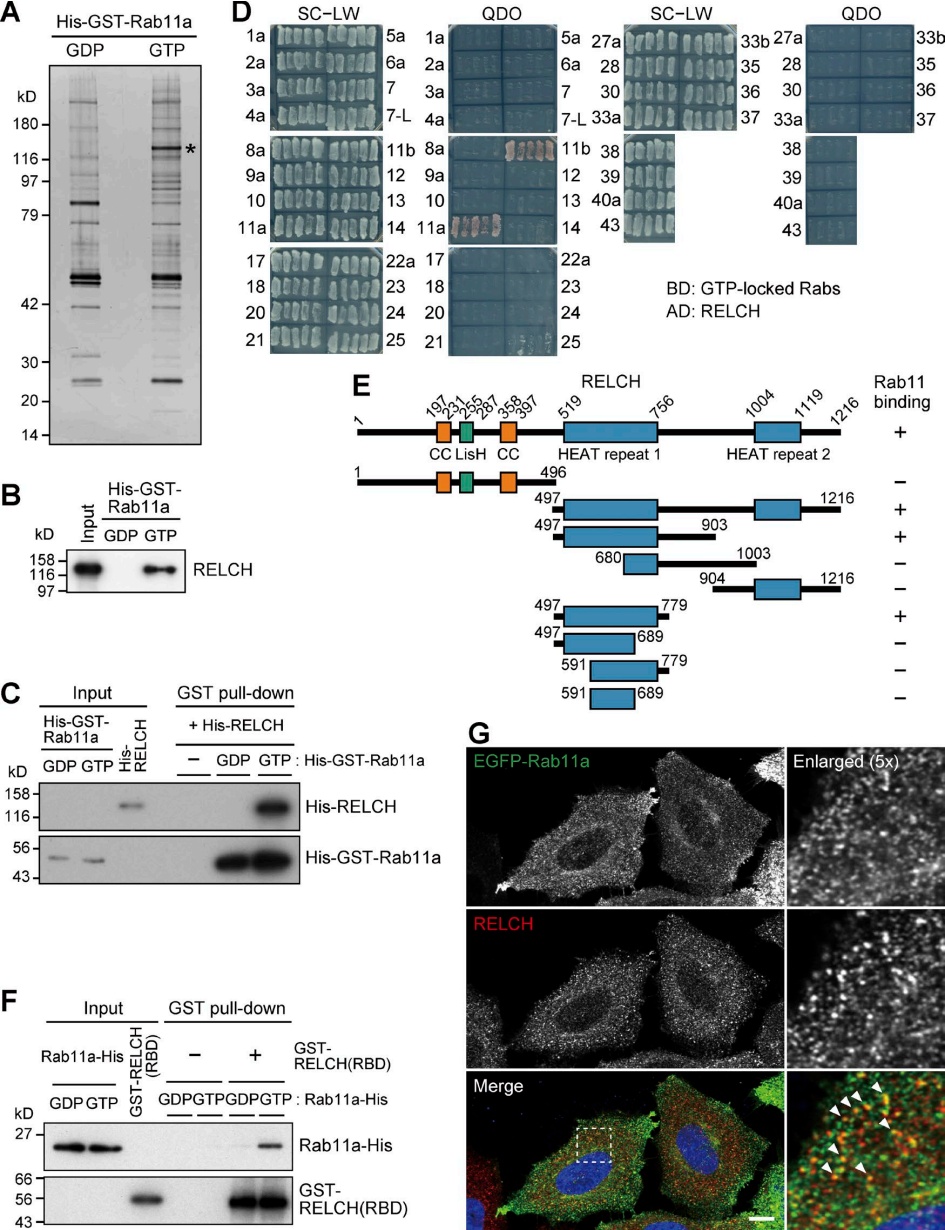
**Identification of RELCH as a Rab11-interacting protein. (A)** The bead-bound GST-Rab11a WT or Q70A protein was incubated with a mouse brain lysate and GDP or GTP, respectively. The samples were separated on 4–12% gradient gels and silver-stained. The band with the asterisk was analyzed by performing mass spectrometry. **(B)** The pulled-down samples shown in A were immunoblotted with a RELCH antibody. **(C)** In vitro pulldown assay using purified recombinant RELCH and GST-Rab11-GTP or -GDP. The samples were immunoblotted with RELCH and GST antibodies. **(D)** Yeast two-hybrid assay using RELCH and GTP-locked Rabs. Yeast cotransformed with Gal4AD (AD) and Gal4BD (BD) plasmids encoding RELCH and Rab-GTP, respectively, was grown on SC−LW plates. Five independent colonies were selected and restreaked on SC−LW and QDO plates. Growth on the QDO plate indicates an interaction between the two proteins. **(E)** The interaction between Rab11 (Q70A) and a series of RELCH deletion mutants was tested by performing a yeast two-hybrid assay. The original data are shown in Fig. S1 A. **(F)** In vitro binding assay using the GST-tagged RBD of RELCH (GST-RELCH [RBD]) and C-terminal His-tagged Rab11a S25N or Q70A loaded with GDP or GTP, respectively. The samples were immunoblotted using Rab11 and GST antibodies. **(G)** HeLa cells expressing EGFP-Rab11a were immunostained with the RELCH antibody. The nuclei were stained with DAPI (blue). The arrowheads indicate RELCH-positive structures overlapped with EGFP-Rab11a–positive structures. Bar, 10 µm.

We performed a GST pulldown assay to identify novel Rab11 binding proteins. A specific interacting protein of ∼130 kD was obtained by incubating mouse brain lysate with GTP-loaded Rab11a (Fig. 1, A and B). The mass spectrometry analysis identified seven peptides corresponding with KIAA1468 (also called the Institute of Physical and Chemical Research cDNA 2310035C23 gene). This protein possesses the Lis1 homology (LisH) domain, coiled-coil (CC) domains, and HEAT repeat motifs (Fig. 1 E). Hereinafter, this protein is designated RELCH (Rab 11–binding protein containing LisH, CC, and HEAT repeats). The direct interaction between RELCH and GTP-bound Rab11 was confirmed using recombinant proteins (Fig. 1 C). To assess the RELCH-binding specificity among the Rab family proteins, we performed a yeast two-hybrid assay. RELCH bound Rab11a and Rab11b and weakly bound Rab25 but did not bind the other 33 Rab proteins (Fig. 1 D). According to a two-hybrid assay using serial deletion mutants of RELCH, the region between residues 497 and 779 containing the first HEAT repeat motif was necessary for the binding of RELCH to Rab11 (Figs. 1 E and S1, A and B). Furthermore, we tested this binding in vitro using a GST-fused 497–779 fragment of RELCH and GDP- or GTP-bound His6×-tagged Rab11a. The fragment specifically bound Rab11a-GTP (Fig. 1 F). By performing immunofluorescence microscopy, we observed that RELCH colocalized with Rab11- and transferrin receptor (TfnR)-positive REs but not with the early/sorting endosomal protein EEA1, the TGN protein p230, or the late endosome (LE)/lysosome proteins cation-dependent mannose-6-phosphate receptor (CD-MPR) and Lamp2 (Figs. 1 G and S1 C). These results indicate that RELCH specifically binds Rab11-GTP.

### Identification of OSBP as a RELCH-interacting protein

**Figure 2.**
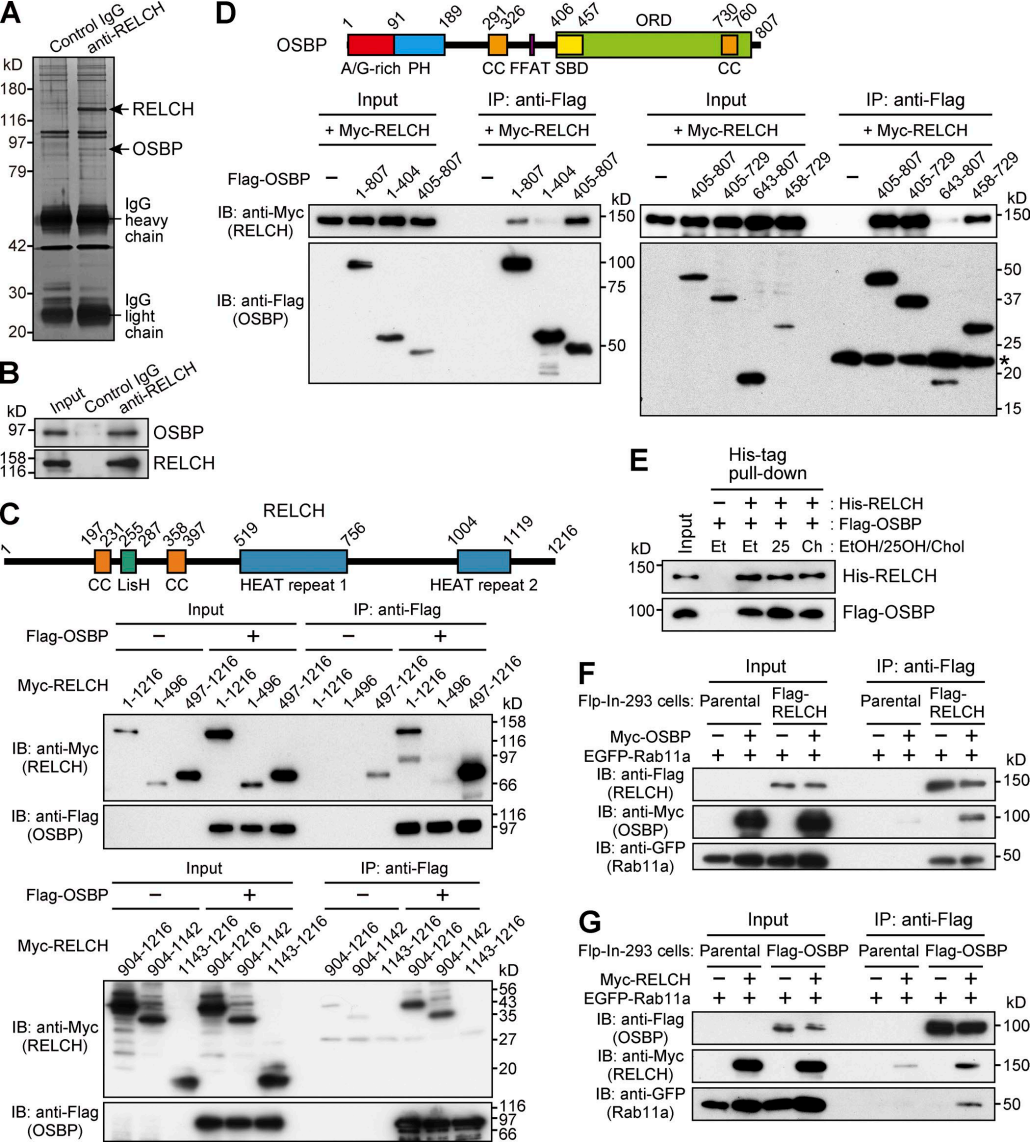
**OSBP is a RELCH-binding protein. (A and B)** Protein G Sepharose beads bound to control IgG or the RELCH antibody were incubated with a mouse brain lysate. The precipitated samples were analyzed by SDS-PAGE and silver staining and further analyzed by mass spectrometry (A). The mass spectrometry analysis identified RELCH and OSBP, which are indicated by the arrows. The precipitated samples were analyzed by immunoblotting (IB) using the OSBP and RELCH antibodies (B). **(C and D)** The lysates of HEK293FT cells coexpressing a series of Flag-tagged OSBP and Myc-tagged RELCH fragments were immunoprecipitated (IP) using a Flag antibody. The samples were immunoblotted with the Myc and Flag antibodies. SBD, sterol-binding domain. In total, 0.5% and 1.5% of the input samples were loaded in upper and lower panels, respectively (C). The asterisk indicates the nonspecific bands (D). **(E)** A His-tag pulldown assay was performed using His-RELCH and Flag-OSBP in the presence of solvent (ethanol, EtOH), 25-OH, or cholesterol (Chol). The samples were immunoblotted with the His and Flag antibodies. **(F and G)** The Flag-, EGFP-, and Myc-tagged proteins were coexpressed in the Flp-In–293 cells. The lysates were immunoprecipitated using a Flag antibody, and the samples were immunoblotted with the EGFP, Myc, and Flag antibodies.

Because RELCH is a Rab11 effector protein, we hypothesized that the depletion of RELCH could cause defects in protein trafficking in the endocytic recycling or exocytic transport pathways, which have been previously reported in studies using cells expressing a Rab11 dominant-negative mutant (Ullrich et al., 1996; Chen et al., 1998) or Rab11-depleted cells (Takahashi et al., 2012). To verify this hypothesis, we performed fluorescence-labeled transferrin internalization/recycling and exocytic vesicular stomatitis virus (VSVG) transport assays (Diao et al., 2008; Linford et al., 2012). However, neither transferrin endocytosis nor recycling defects were observed in the RELCH-depleted cells (Fig. S2, A and B). In the exocytic VSVG transport assay, compared with the Rab11a/b-depleted cells, we observed a minor effect in the RELCH-depleted cells (Fig. S2, A and C). Furthermore, we performed SDS-PAGE to examine the mobility of the transmembrane cargo proteins exiting the TGN because a previous study reported that certain cargo proteins from Rab11a-deficient small intestinal cells had slower mobility than proteins from control cells as a result of abnormal glycosylation, which likely reflects decelerated protein export from the TGN (Sobajima et al., 2014). In the Rab11-depleted HeLa cells, we also observed slower SDS-PAGE mobility in TfnR, Lamp2, and TGN46 (Fig. S2, A and D). In contrast, no obvious differences were observed in the mobility of these glycoproteins between the RELCH-depleted cells and the control cells (Fig. S2, A and D). These results indicate that the RELCH–Rab11 complex plays an unknown role. Therefore, to obtain insight into its function, we attempted to identify the RELCH-interacting protein. Immunoprecipitation with RELCH from mouse brain lysate demonstrated the coprecipitation of an ∼90-kD protein. Mass spectrometry analysis revealed that this protein was identical to OSBP (Fig. 2, A and B). To identify the region of RELCH that binds OSBP, Flag-tagged OSBP and Myc-tagged RELCH serial deletion mutants were coexpressed in HEK293FT cells, and the proteins were immunoprecipitated using an anti-Flag antibody. Full-length RELCH and C-terminal region fragments containing the second HEAT repeat motif (residues 497–1,216, 904–1,216, and 904–1,142) were coprecipitated with OSBP, but the N-terminal region (1–496) and a C-terminal mutant (1,143–1,216) were not (Fig. 2 C). These data indicate that OSBP binds the HEAT repeat 2–containing region of RELCH. We further examined the RELCH-binding region of OSBP. The full-length and C-terminal regions (405–807, 405–729, and 458–729) of OSBP coprecipitated with RELCH, but the N-terminal region (1–404), C-terminal CC, and a part of ORD (643–807) did not. These data indicate that RELCH binds the C-terminal region of OSBP, which contains the ORD (Fig. 2 D). The binding experiments using the purified proteins confirmed the presence of a direct interaction between RELCH and OSBP (Fig. 2 E). Moreover, the immunoprecipitation experiments using cell lysates coexpressing epitope-tagged Rab11a, RELCH, and OSBP showed that these proteins could form a ternary complex (Fig. 2, F and G).

### RELCH links Rab11 to OSBP for RE relocation to the TGN area

**Figure 3.**
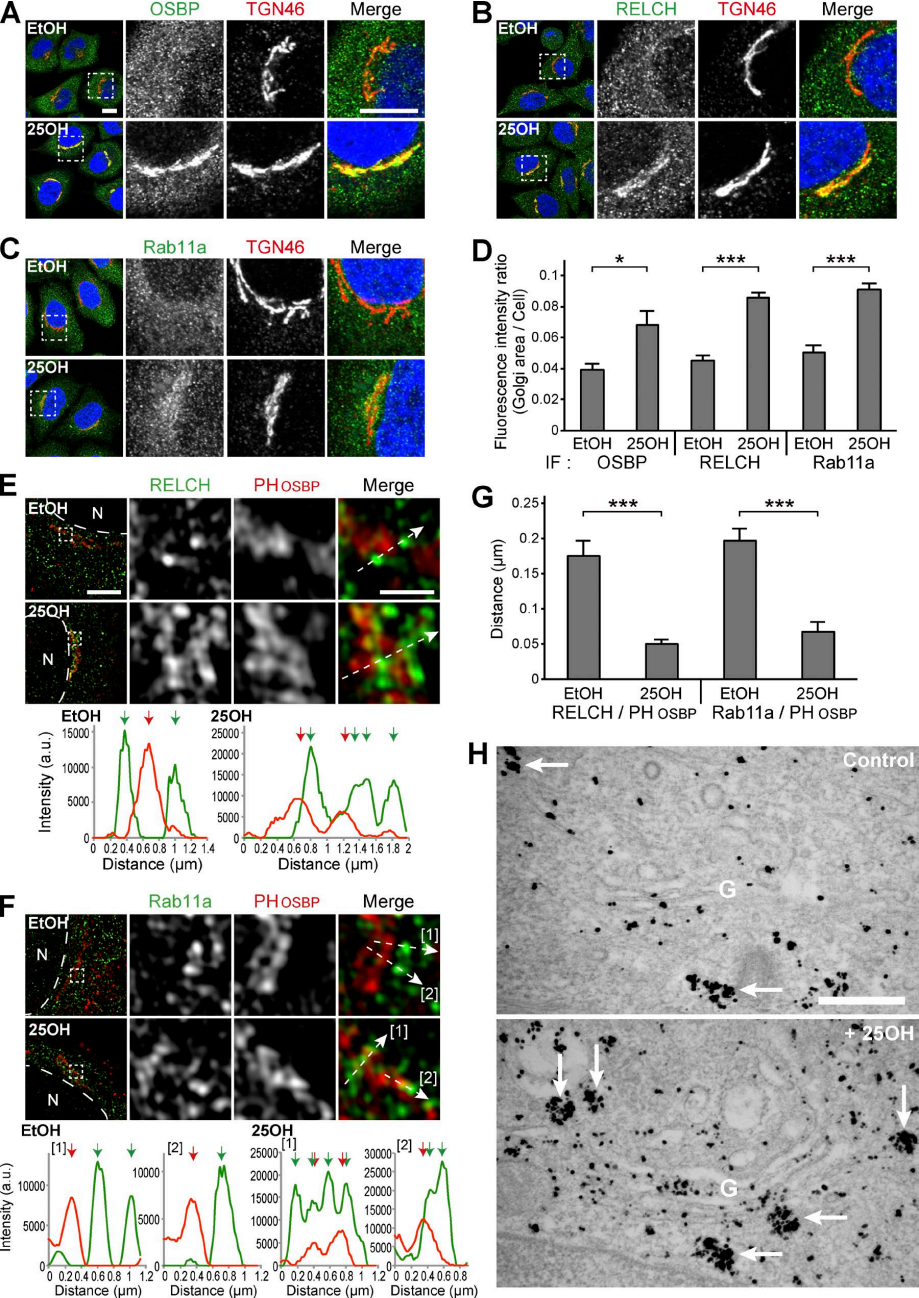
**Localization of RELCH, OSBP, and Rab11 after 25-OH treatment. (A–D)** HeLa cells were incubated with 6.2 µM 25-OH or solvent (ethanol, EtOH) for 24 h and immunostained with antibodies against TGN46 and OSBP (A), RELCH (B), or Rab11a (C). The nuclei were stained with DAPI (blue). The enlarged images show the TGN area. Bar, 10 µm. **(D)** Quantification of the ratio of the fluorescence intensity in the Golgi area to the intensity in the whole intracellular area (*n* = 10–25 cells). **(E–G)** HeLa cells transfected with the Flag-tagged PH domain of OSBP (PH_OSBP_) were incubated with EtOH or 25-OH for 24 h before analysis. The cells were immunostained with antibodies against Flag and either RELCH (E) or Rab11a (F) and imaged by SR-SIM. The fluorescence intensity profiles of PH_OSBP_ and either RELCH or Rab11a were measured at the position marked by the arrows in the merged insets. The red and green arrows in the graphs indicate the intensity peak of PH_OSBP_ and RELCH or Rab11a, respectively. N, nucleus. Bars: (main images) 5 µm; (enlarged images) 1 µm. **(G)** Quantification of the distance between the intensity peak of PH_OSBP_ and RELCH or Rab11a (*n* = 15–34 cells). **(H)** Immunoelectron microscopy of HeLa cells treated with 25-OH and nontreated control cells after the EGFP-Rab11a transfection. Silver-enhanced gold particles were used to label the EGFP-Rab11a–positive vesicles and tubules (arrows). Bar, 500 nm. Data are expressed as means ± SEM. Significance was calculated by performing two-tailed Student’s *t* tests (*, P < 0.05; ***, P < 0.001). IF, immunofluorescence.

**Figure 4.**
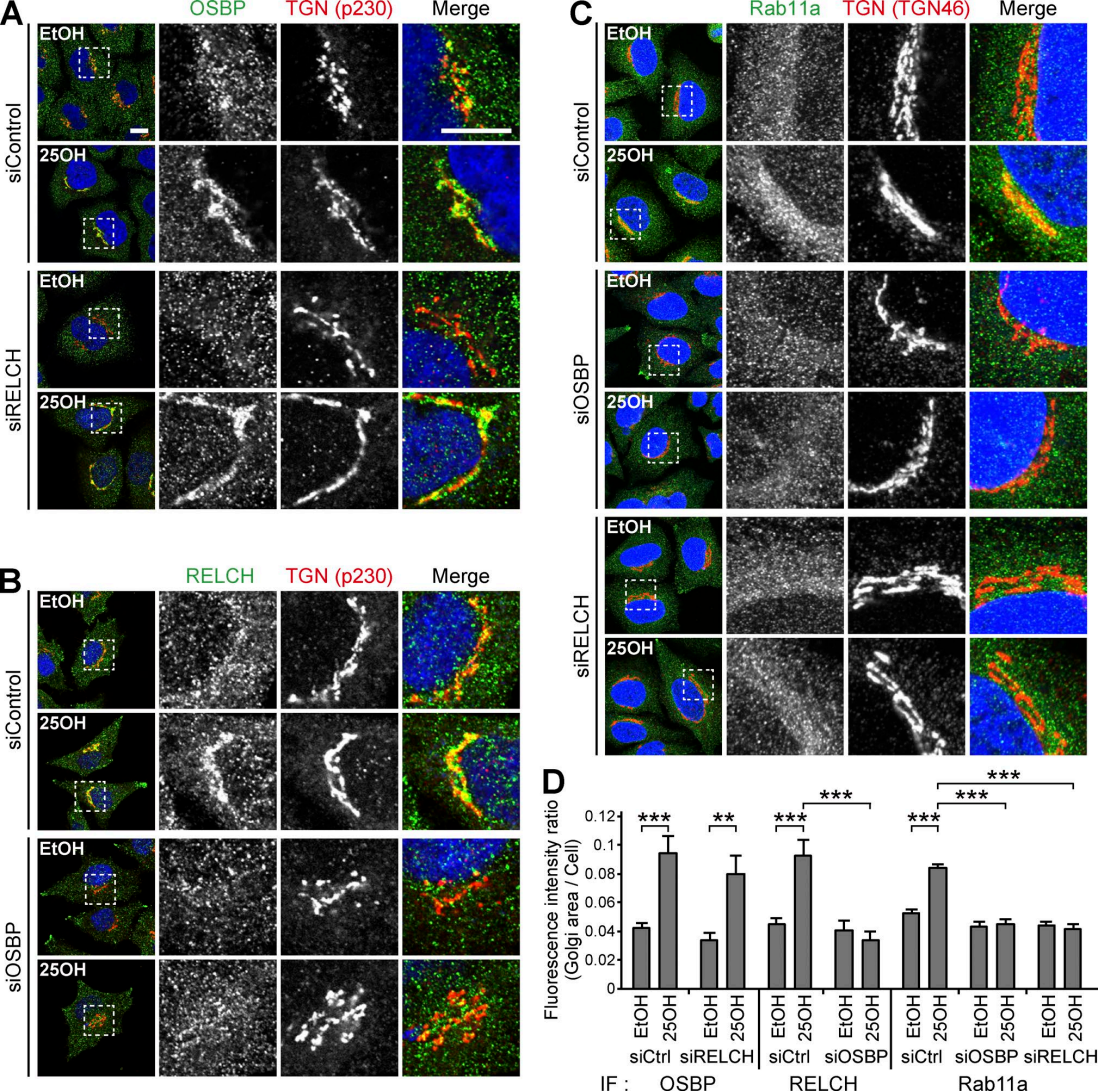
**Interdependence among RELCH, OSBP, and Rab11 in TGN localization after 25-OH treatment. (A–C)** siRNA-transfected HeLa cells were incubated with solvent (ethanol, EtOH) or 25-OH for 24 h before analysis. The cells were immunostained with antibodies against OSBP (A), RELCH (B), and Rab11a (C). The TGN was labeled with the TGN46 or p230 antibodies. The nuclei were stained with DAPI (blue). The enlarged images show the TGN area. Bar, 10 µm. **(D)** Quantification of the ratio of the fluorescence intensity in the Golgi area to the intensity in the intracellular area (*n* = 10–39 cells). Data are expressed as means ± SEM. Significance was calculated by performing two-tailed Student’s *t* tests (**, P < 0.01; ***, P < 0.001). IF, immunofluorescence.

OSBP is a cholesterol transfer or cholesterol-sensing protein that functions at the TGN (Wang et al., 2005; Mesmin et al., 2013). In cells treated with 25-hydroxycholesterol (25-OH), which may mimic a cholesterol-starved state, OSBP bound 25-OH and translocated to the TGN from the cytoplasm through its pleckstrin homology (PH) domain (Ridgway et al., 1992). Under this condition, RELCH and Rab11 also translocated to the juxtanuclear TGN area (Fig. 3, A–D; and Fig. S3 A). Furthermore, superresolution structured illumination microscopy (SR-SIM) revealed that the RELCH- and Rab11-positive endosomes were positioned close to, but were distinguishable from, the PH_OSBP_-labeled TGN after the 25-OH treatment (Fig. 3, E–G). By performing immunoelectron microscopy, we confirmed that the gold-labeled Rab11a-positive structures and Golgi apparatus were located close to each other after the 25-OH treatment (Fig. 3 H). To better understand the role played by these proteins in RE relocation close to the TGN area, we examined the effect of 25-OH treatment in OSBP-, RELCH-, and Rab11a/b-depleted cells. These proteins were depleted by >90% using specific siRNAs (Fig. S2 A). RELCH depletion did not affect the OSBP translocation to the TGN after the 25-OH treatment (Fig. 4, A and D). In contrast, 25-OH–induced RELCH translocation to the TGN area was diminished in the OSBP-depleted cells (Fig. 4, B and D). These data suggest that 25-OH–induced RELCH translocation to the TGN area is dependent on OSBP. Moreover, translocation of the Rab11a-positive endosomes to the TGN area in response to the 25-OH treatment was diminished in the OSBP- and RELCH-depleted cells (Fig. 4, C and D). In the Rab11a/b-depleted cells, translocation of OSBP and RELCH to the TGN area was strongly induced regardless of 25-OH treatment (Fig. S3, B, C, and E). Although the mechanism spontaneously targeting the OSBP and RELCH to the TGN area in the absence of Rab11 is unknown, these data indicate that Rab11 determines the localization of RELCH on the RE. In addition, the triple knockdown of Rab11a/Rab11b/OSBP blocked translocation of RELCH to the TGN (Fig. S3, D and E). Altogether, these data indicate that RELCH links Rab11 to OSBP for RE relocation to the TGN area.

### Intracellular cholesterol distribution was altered in RELCH-, OSBP-, and Rab11-depleted cells

**Figure 5.**
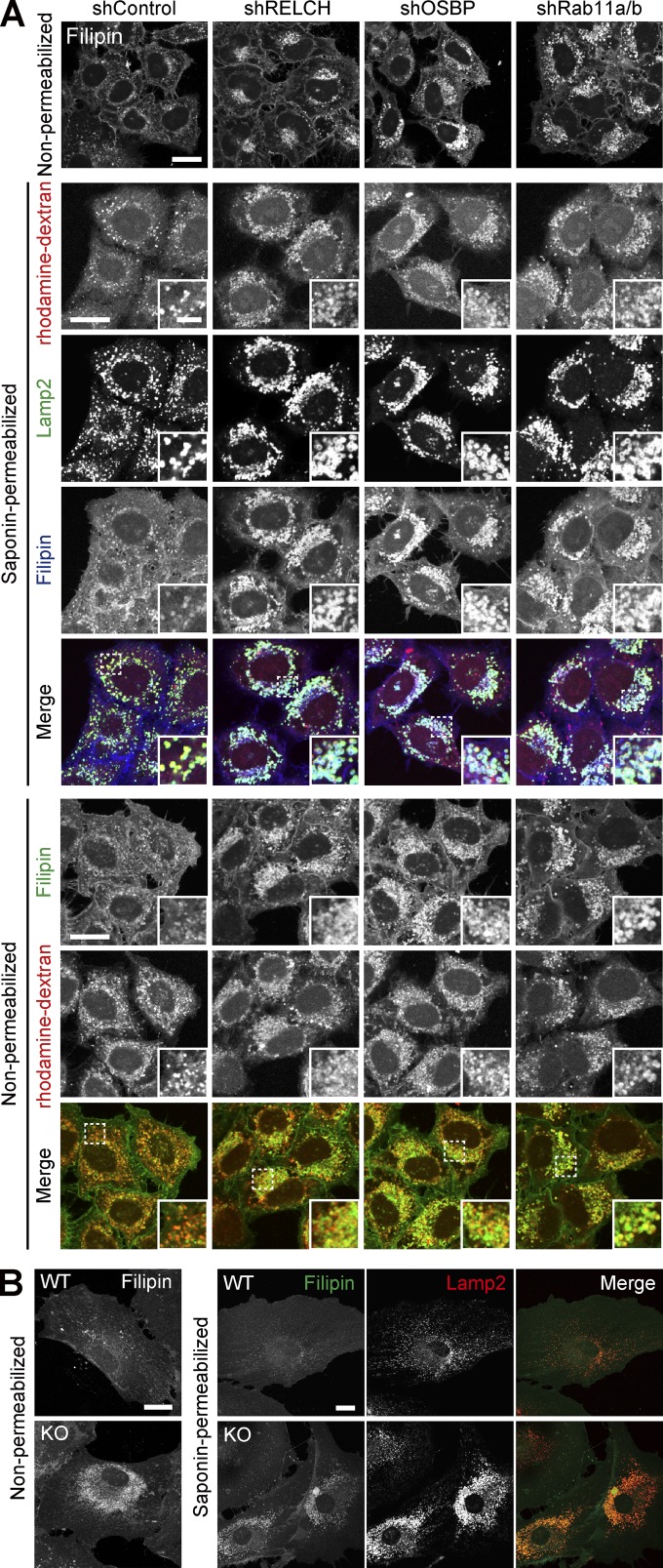
**Cholesterol accumulation in LEs/lysosomes after RELCH, OSBP, or Rab11 depletion. (A)** The lysosomes were labeled with rhodamine-dextran in the shRNA-treated cells. The cells were stained with Filipin under the nonpermeabilized condition and Filipin and Lamp2 under the permeabilized condition. **(B)** Mouse tail–tip fibroblasts derived from WT and RELCH KO mice were stained with the Lamp2 antibody and/or Filipin. Bars: (main images) 20 µm; (insets) 2 µm.

**Figure 6.**
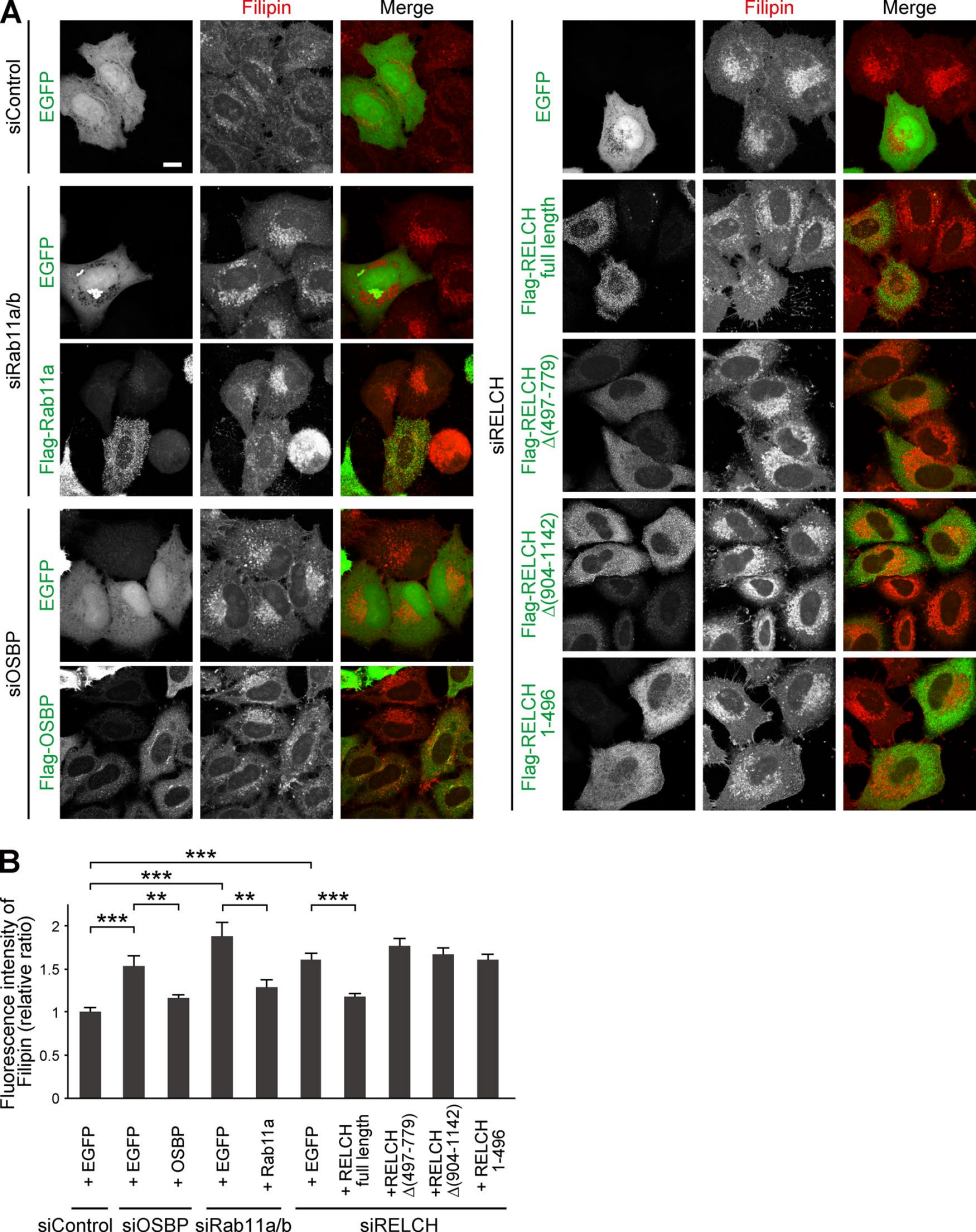
**Expression of RELCH, OSBP, or Rab11 rescues the effect on cholesterol accumulation in the RELCH-, OSBP-, or Rab11-depleted cells. (A)** HeLa cells transfected with the indicated siRNA and plasmids were costained with Filipin and the Flag antibody. Bar, 10 µm. **(B)** Quantification of the fluorescence intensity of Filipin in the plasmid-transfected cells (relative to siControl + EGFP cells; *n* = 18–89 cells). Data are expressed as means ± SEM. Significance was calculated by performing two-tailed Student’s *t* tests (**, P < 0.01; ***, P < 0.001).

**Figure 7.**
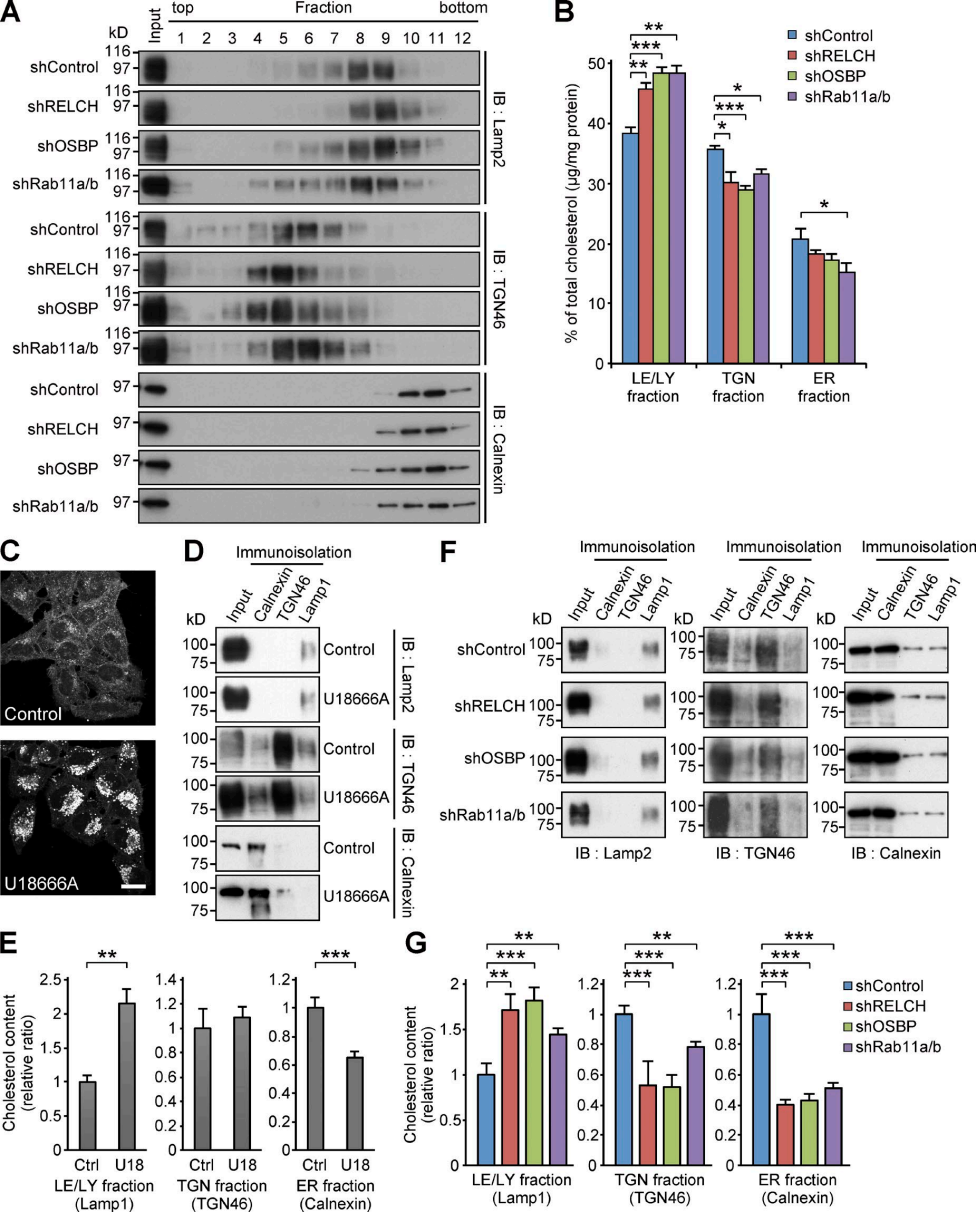
**RELCH, OSBP, and Rab11 depletion results in less cholesterol accumulation in the TGN and ER. (A and B)** The homogenates from the shRNA-expressing HeLa cells were fractionated using a Histodenz step density gradient. **(A)** The fractions were immunoblotted (IB) with antibodies against TGN46, calnexin, and Lamp2. **(B)** Percentages of cholesterol (µg/mg protein) in the TGN (fractions 4–6), LEs/lysosomes (LE/LY; 7–9), or ER (10 and 11) in the total fractions are shown in the bar graph. **(C)** HeLa cells were treated with 2 µg/ml U18666A for 16 h and stained with Filipin. Bar, 20 µm. **(D–G)** Immunoisolation of ER, TGN, and LE/lysosome from the PNS derived from the U18666A-treated (D and E) or RELCH-, Rab11a/b-, and OSBP-depleted cells by shRNAs (F and G). ER, TGN, and LE/lysosome membranes were isolated using calnexin, TGN46, and Lamp1 antibodies, respectively. **(D and F)** The isolated samples were immunoblotted with calnexin, TGN46, and Lamp2 antibodies. **(E and G)** Quantification of the cholesterol content in the isolated membranes (relative to the control samples). Data are expressed as means ± SEM from at least three independent experiments. *, P < 0.05; **, P < 0.01; ***, P < 0.001 (relative to the control; two tailed Student’s *t* tests). Representative data from three independent experiments are shown in A, D, and F.

**Figure 8.**
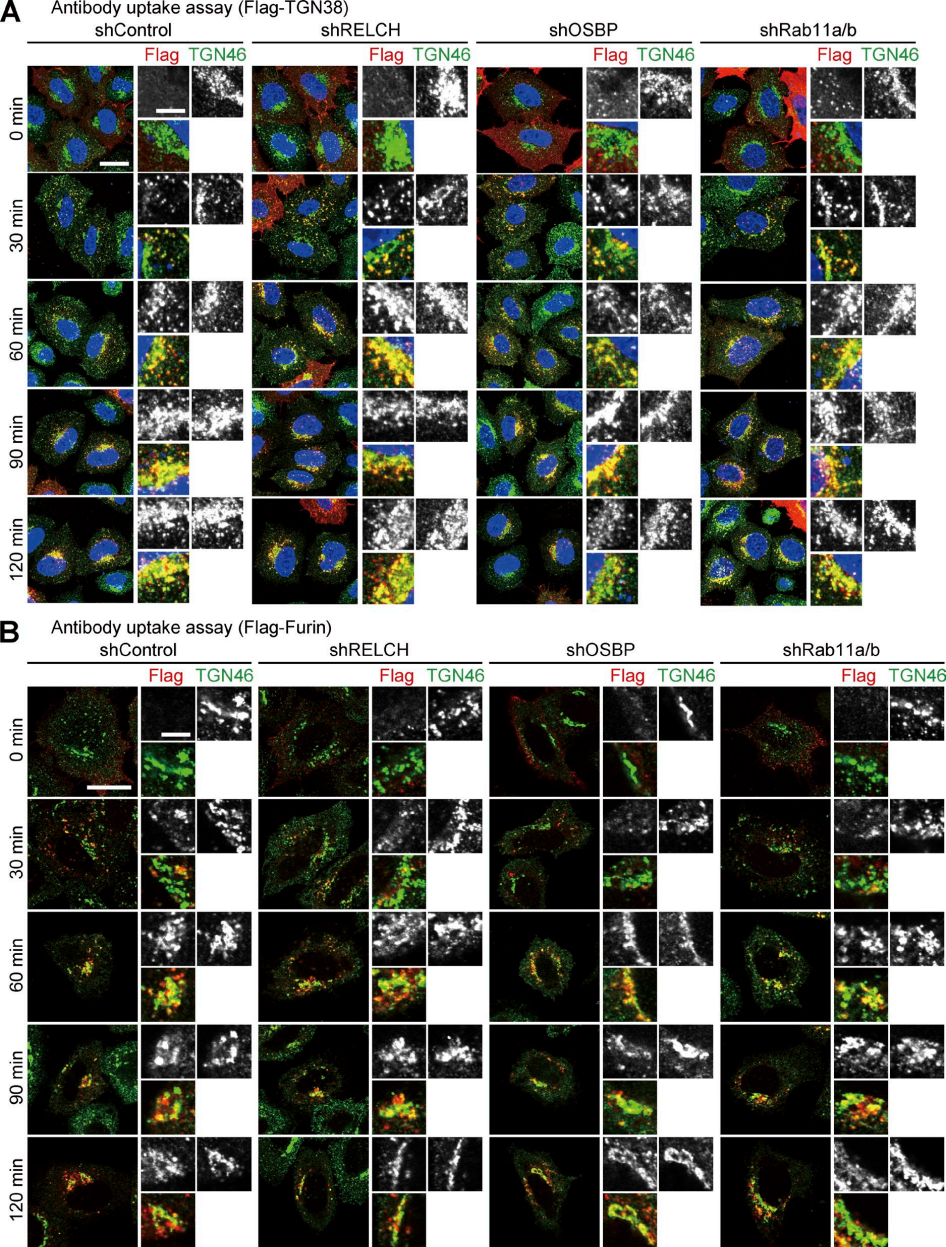
**Retrograde transports of TGN38 and Furin in the RELCH-, OSBP-, and Rab11-depleted HeLa cells. (A and B)** Antibody uptake assay in HeLa cells stably expressing the indicated shRNAs. The cells transfected with Flag-tagged TGN38 (A) or Furin (B) were incubated with a Flag antibody on ice for 45 min and then chased at 37°C in growth medium. The cells were fixed at the indicated times and immunostained with the Flag and TGN46 antibodies. The nuclei were stained with DAPI (blue). Bars: (main images) 20 µm; (enlarged images) 5 µm.

Because RELCH directly bound OSBP and was translocated to the TGN area in an OSBP-dependent manner (Figs. 2, 3, 4, and S3), we hypothesized that RELCH and Rab11 function with OSBP in cholesterol transport. Therefore, we examined the subcellular distribution of cholesterol in RELCH-, OSBP-, and Rab11a/b-depleted cells. Compared with the control cells, cholesterol accumulated in the LEs/lysosomes in depleted cells as indicated by Filipin or BODIPY-cholesterol staining (Figs. 5 A and S4, A and B). However, no obvious accumulation of cholesterol was observed in the TGN area or Rab11- and TfnR-positive REs using fluorescence microscopy (Fig. S4, C–E). Moreover, compared with the control cells, in the RELCH-, Rab11a/b-, and OSBP-depleted cells, the LEs/lysosomes were enlarged, positioned at the juxtanuclear region, and resembled the LEs/lysosomes in fibroblasts derived from patients with lipid storage disorders such as Niemann–Pick syndrome type C (Sokol et al., 1988). Cholesterol accumulation in enlarged and clustered LEs/lysosomes at the juxtanuclear region was also observed in fibroblasts derived from RELCH knockout (KO) mice (Figs. 5 B and S2 E). Subsequently, we performed rescue experiments by expressing Flag-tagged OSBP, Rab11, or RELCH. The expression of these proteins rescued the effect on cholesterol accumulation in the OSBP-, Rab11a/b-, and RELCH-depleted cells (Fig. 6, A and B). In contrast, the expression of the RELCH mutants Δ (497–779), Δ (904–1,143), and (1–496), which fail to bind Rab11, OSBP, and both, respectively, did not rescue the effect on cholesterol accumulation in the LEs/lysosomes in the RELCH-depleted cells (Fig. 6, A and B). These data suggest that Rab11, RELCH, and OSBP function together in cholesterol transport. To examine the cholesterol distribution in the organelles in greater detail, we used a subcellular fractionation technique with OSBP-, RELCH-, and Rab11a/b-depleted cells. The cell homogenates were separated by density gradient centrifugation, and the cholesterol content in each fraction was measured. The TGN membrane was marked by TGN46 and concentrated in fractions 4–6, the LEs/lysosomes were marked by Lamp2 and concentrated in fractions 7–9, and the ER was marked by calnexin and concentrated in fractions 10–11 (Fig. 7 A). The cholesterol content was increased in the LE/lysosome fractions of the RELCH-, OSBP-, and Rab11a/b-depleted cells (Fig. 7 B). In contrast, compared with the control cells, the cholesterol levels in the TGN and ER fractions were slightly decreased in the RELCH-, OSBP-, and Rab11a/b-depleted cells (Fig. 7 B). However, it is difficult to exclude entirely the contamination of other organelle membranes between fractions using this method; therefore, we further measured the cholesterol content in the ER, TGN, and LE/lysosome membranes, which were isolated using antibodies against calnexin, TGN46, and Lamp1, respectively. First, we measured the cholesterol content using membranes isolated from cells treated with U18666A, which is an inhibitor of the NPC1 protein (Lange et al., 2000; Lu et al., 2015). Cholesterol content in the LEs/lysosomes from the U18666A-treated cells was increased, which is consistent with the fluorescence microscopy observation (Fig. 7, C–E). This result indicates that the immunoisolation technique can be used to measure cholesterol content in the organelle membrane. Then, we measured the cholesterol content in membranes isolated from RELCH-, OSBP-, and Rab11a/b-depleted cells. Cholesterol content in the LEs/lysosomes from the RELCH-, OSBP-, and Rab11a/b-depleted cells was higher than that in the control cells. However, cholesterol content in the TGN and ER membranes from the knockdown cells was lower than that in the control cells (Fig. 7, F and G). To determine whether cholesterol accumulation in the LEs/lysosomes was caused by defects in vesicle-mediated transport, we performed a retrograde transport assay using Flag-tagged TGN38 and Furin, which traffics from the PM to the TGN through the RE or LE/lysosome, respectively (Shin et al., 2004; Chia et al., 2011). The trafficking of TGN38 and Furin from the PM to the TGN was not altered in Rab11-, RELCH-, and OSBP-depleted cells (Fig. 8). Therefore, these data suggest that RELCH, OSBP, and Rab11a/b are involved in nonvesicular cholesterol transport from the endocytic compartment to the TGN and ER; thus, cholesterol accumulated in the lysosomes.

### RELCH, OSBP, and Rab11 mediate cholesterol transfer between the RE and TGN in vitro

**Figure 9.**
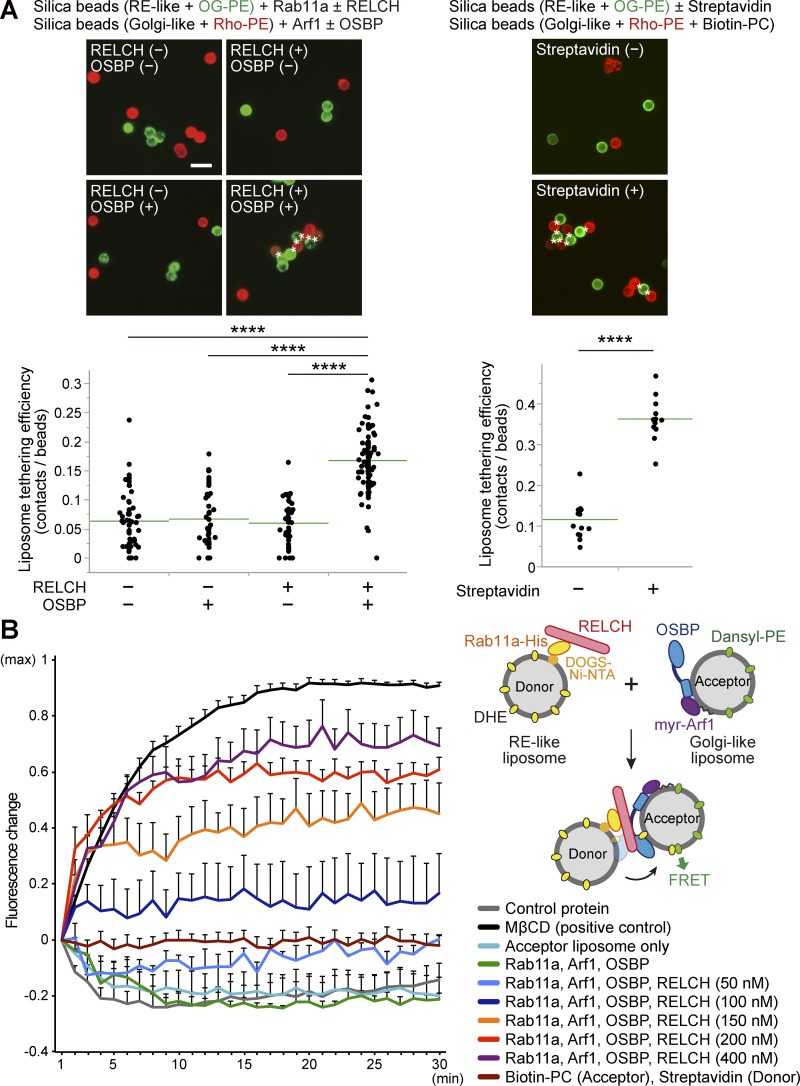
**RELCH controls the OSBP-dependent cholesterol transfer from the RE-like membrane to the Golgi-like membrane via tethering activity. (A)** Bead-based liposome tethering assay between Golgi- and RE-like liposomes containing rhodamine-PE (Rho-PE; red) and OG-PE (green), respectively. The protein-bound beads (the binding of myr-Arf1, Rab11a, OSBP, and RELCH is demonstrated in Fig. S5 [A–D]) were mixed and imaged under a fluorescence microscope (top left). In the top right panel, the tethering assay using the streptavidin-His protein and biotin-PC–containing liposomes is shown. The asterisks indicate the contacts between two beads. The liposome tethering efficiency was calculated as the ratio of the number of contacts to the total number of beads. The data were collected from randomly selected fields per group. Representative examples of the counts and calculations are shown in Fig. S5 G. The results are presented in the dot-plot graphs (bottom). The green lines indicate the mean of each group. ANOVA, Wilcoxon, and Kruskal–Wallis tests were performed (****, P < 0.0001). Bar, 10 µm. **(B)** Lipid transfer assay between RE-like donor and Golgi-like acceptor liposomes containing DHE and dansyl-PE, respectively. Recombinant proteins were bound to each type of liposome (right). The transfer reaction was initiated by mixing the two types of protein-bound liposomes. MβCD was used as a positive control to determine the maximum FRET signal. Maltose binding protein was used as a control protein. The data from each experiment were corrected by setting the initial and maximum values to 0 and 1, respectively, and are presented in the line graph (left). Data are expressed as means ± SEM from at least three independent experiments.

Several studies have indicated that lipid transfer between organelle membranes occurs at MCSs and is mediated by a tethering mechanism (Prinz, 2014; Eisenberg-Bord et al., 2016). Therefore, we hypothesized that RELCH, OSBP, and Rab11 might be tethered between the TGN and RE membranes. Consistently, our results showed that REs accumulated around the TGN area in the 25-OH–treated cells in an OSBP- and RELCH-dependent manner, suggesting that these proteins function in tethering between the TGN and RE (Fig. 4). To further examine this mechanism, we performed an in vitro assay using purified recombinant proteins and artificial liposomes following a protocol slightly modified from that proposed by Mesmin et al. (2013). First, we confirmed that OSBP bound Golgi-like lipid-coated silica beads prebound with myristoylated Arf1-GTP protein (myr-Arf1(Q71L)-Flag; Fig. S5, A and B). Then, we demonstrated that RELCH bound silica beads coated with an RE-like nickel-containing lipid (DOGS-NTA(Ni)) prebound with His6×-tagged Rab11a-GTP (Rab11a(Q70A)-His; Fig. S5, C and D). The binding of these proteins to the lipids was further confirmed using a liposome flotation or sedimentation assay (Fig. S5, E and F). Then, we mixed Arf1-GTP–bound beads coated with a rhodamine-phosphatidylethanolamine (PE)–labeled Golgi-like lipid and Rab11-GTP-bound beads coated with an Oregon green 488–DHPE (OG-PE)–labeled RE-like lipid in the presence or absence of OSBP and RELCH. Compared with reactions lacking both proteins or reactions with only RELCH or OSBP, aggregate formation between the Golgi-like and RE-like liposomes occurred more frequently after adding both RELCH and OSBP (Fig. 9 A, left, and Fig. S5 G). Aggregation between the streptavidin-bound liposomes and biotin-conjugated phosphatidylcholine (PC)–containing liposomes was also observed (Fig. 9 A, right). Therefore, these data suggest that RELCH tethers between OSBP-bound and Rab11-bound membranes.

Finally, we examined cholesterol transfer using an in vitro system. Golgi-like liposomes containing dansyl-PE and RE-like liposomes containing dehydroergosterol (DHE) were mixed, and the fluorescence resonance energy transfer (FRET) between dansyl and DHE was measured, which revealed sterol transfer from the RE-like liposomes to the Golgi-like liposomes. After adding Rab11a, Arf1, OSBP, and different concentrations of RELCH, the DHE transfer occurred in a RELCH dose-dependent manner, but no significant DHE transfer activity was observed in the reactions lacking RELCH (Fig. 9 B). However, DHE transfer activity between the streptavidin-bound liposomes and biotin-PC liposomes was not observed (Fig. 9 B), indicating that the cholesterol transfer did not simply reflect the aggregation of liposomes. These results suggest that RELCH functions in membrane tethering and promotes OSBP-mediated cholesterol transfer between Rab11-bound RE and OSBP-bound Golgi-like membranes.

## Discussion

We identified that RELCH is a Rab11-GTP binding protein and revealed the interaction between RELCH and OSBP. In cells treated with 25-OH, Rab11-positive REs were gathered around the TGN area in a RELCH- and OSBP-dependent manner, suggesting that membrane tethering between the RE and TGN occurred through the intracellular sterol-sensing mechanism of OSBP. Our experiments using artificial liposomes indicated that the tethering mediated by these proteins is crucial for efficient cholesterol transfer between the membranes.

RELCH contains two HEAT repeat motifs. The HEAT motif comprises α-helical structures and functions in protein–protein interactions (Yoshimura and Hirano, 2016). The first N-terminal HEAT repeat motif in RELCH likely binds Rab11-GTP in a manner similar to that of other Rab proteins that preferentially bind α-helices (Fig. 1, E and F; and Fig. S1, A and B; Khan and Ménétrey, 2013). In addition, the second C-terminal HEAT repeat motif in RELCH interacts with OSBP (Fig. 2 C). In a previous study, OSBP was shown to directly interact with the protein phosphatase 2A (PP2A) complex, which has subunits containing HEAT repeats or HEAT-like α-helical structures (Wang et al., 2008). Although the precise region of each PP2A subunit necessary for OSBP binding was not examined, the authors speculated that the HEAT repeats in both subunits are involved in the interaction. Therefore, RELCH may interact with OSBP via a similar mechanism as PP2A.

After the 25-OH treatment, RELCH was strongly recruited around the TGN area in an OSBP-dependent manner (Figs. 3 and 4), suggesting that the interaction between the two proteins is enhanced at the TGN, although the precise mechanism of this enhancement remains unknown. However, we can exclude the possibility that the sterol-binding state of OSBP affects its binding to RELCH because the in vitro pulldown experiment using purified proteins indicated that RELCH binds OSBP regardless of sterol binding (Fig. 2 E). We speculated that the proteins are posttranslationally modified at the TGN and that these modifications may strengthen the interaction between these proteins. Consistently, OSBP is phosphorylated at the TGN by Golgi-localized protein kinase D (Nhek et al., 2010). Moreover, RELCH possesses numerous phosphorylation sites (Hornbeck et al., 2015); therefore, determining whether the phosphorylation of these proteins controls their binding could be interesting.

Filipin or BODIPY-cholesterol staining indicated that cholesterol accumulated in the LEs/lysosomes in the RELCH-, OSBP-, and Rab11-depleted cells (Figs. 5 and S4). Furthermore, the TGN and ER cholesterol contents in the RELCH-, OSBP-, and Rab11-depleted cells were reduced compared with those in the control cells (Fig. 7). We did not detect any obvious alteration in the vesicular trafficking from the LE/lysosome or RE to the TGN in these protein-depleted cells (Fig. 8). In addition, the in vitro reconstitution experiments showed that RELCH promotes OSBP-dependent nonvesicular cholesterol transport to the TGN-like membrane (Fig. 9). Altogether, these results suggest that the Rab11–RELCH–OSBP complex is involved in cholesterol transport from the LE/lysosome to the TGN and ER. However, the LE/lysosome-TGN MCSs at which lipid transfer occurs have not been identified. Nevertheless, in previous studies, the MCS formation between the LE/lysosome and ER was observed, but whether cholesterol is directly transferred from the LE/lysosome to the ER was not determined (Rocha et al., 2009; Alpy et al., 2013; Raiborg et al., 2015). Because the RELCH and Rab11 localization is limited to the RE or TGN-adjacent area (Figs. 1 G and S1 C), the Rab11–RELCH–OSBP complex is unlikely to directly mediate the cholesterol transport from the LE/lysosome to the TGN or ER. The most likely scenario is that cholesterol traffics from the LE/lysosome to the TGN via the RE, and this complex mediates nonvesicular cholesterol transport from the RE to the TGN and, consequently, affects cholesterol transport from the TGN to the ER. However, we did not detect significant cholesterol accumulation in the RE by Filipin or BODIPY-cholesterol staining in the RELCH-, OSBP-, and Rab11-depleted cells (Fig. S4, D and E). Thus, we speculate that cholesterol transiently exports from the LEs/lysosomes and rapidly traffics through the REs, and the undelivered cholesterol from the RE, reflecting defects in the RE–TGN pathway caused by the depletion of RELCH, OSBP, and Rab11, is retrieved to the LEs/lysosomes.

Importantly, Urano et al. (2008) demonstrated that the depletion of three SNARE proteins, i.e., VAMP4, syntaxin6, and syntaxin16, that are involved in vesicular traffic between the early endosome/RE and the TGN (Mallard et al., 2002) inhibited cholesterol transport from the lysosome to the TGN and resulted in the partial inhibition of cholesterol reesterification in the ER. Moreover, the depletion of a subunit of Golgi-associated retrograde protein (GARP), which is a tethering complex that regulates endosome–Golgi retrograde vesicular transport, resulted in cholesterol accumulation in the LEs/lysosomes (Fröhlich et al., 2015). In conjunction with these findings, the results of this study suggest that two different cholesterol transport mechanisms exist in LE/lysosome-RE-TGN trafficking, i.e., a vesicular transport mechanism mediated by GARP and SNARE complexes and a nonvesicular transport mechanism mediated by Rab11–RELCH–OSBP. We assume that the two mechanisms cooperatively function in cholesterol transport from REs to the TGN.

The lipid raft hypothesis highlights the importance of cholesterol content in the TGN (Surma et al., 2012; Guo et al., 2014). Previous findings suggest that sphingolipid/sterol-rich lipid microdomains at the TGN (often considered a lipid raft) are involved in sorting specific sets of cargo proteins, e.g., glycosylphosphatidylinositol (GPI)-anchored protein, from the TGN to the PM (Hansen et al., 2000; Castillon et al., 2013). Interestingly, cells deficient in subunits of the GARP complex exhibit defects in the exit of GPI-anchored protein from the TGN to the PM (Hirata et al., 2015). These findings suggest that the transport defect in GARP-KO cells might reflect a disruption in lipid microdomain formation at the TGN. Therefore, it will be important to address the contribution of Rab11–RELCH–OSBP–mediated nonvesicular cholesterol transport to lipid microdomain formation and lipid microdomain-dependent protein-cargo sorting at the TGN, in addition to vesicular transport mechanisms.

## Materials and methods

### Plasmids

A pFAT2 vector encoding the His-GST-Rab constructs, a pFBT9 vector encoding the Gal4BD-GTP–locked Rab constructs, and a pEGFP-C2 vector encoding the EGFP-fused Rab constructs were generated as previously described (Haas et al., 2005; Yoshimura et al., 2007). Mouse RELCH/KIAA1468 (accession no. NM_173187.3) and mouse Rab11a were PCR amplified from the Mouse 17-d Embryo Marathon Ready cDNA library (Takara Bio Inc.) using KOD DNA polymerase (Toyobo). Then, the cDNA was subcloned into the pSCB vector using the StrataClone Blunt PCR cloning kit (Agilent Technologies). A series of RELCH deletion mutants and a RELCH fragment containing an N-terminal PreScission protease recognition site were generated by PCR using pSCB-RELCH. The RELCH constructs were inserted into the pQE32tev, pFAT2, pACT2, pcDNA3.1-Myc, and pcDNA5/FRT/TO-Flag vectors according to their intended use. C-terminal His-tagged mouse Rab11a S25N and Q70A mutants (GDP- and GTP-locked mutants, respectively) without a stop codon were generated by PCR using pSCB-mouse Rab11a and then ligated into the NcoI and XhoI sites of the pET28c vector. Human OSBP, a C-terminal Flag-tagged human Arf1 Q71L mutant, and yeast NMT1 were PCR amplified from pOTB7-OSBP (4560111; GE Healthcare), pcDNA3.1-Arf1 (Q71L)-HA (10832; Addgene), and pRSF-1-NMT (42578; Addgene), respectively. OSBP, PH_OSBP_ (OSBP_91-189_), and OSBP deletion mutants and mouse Rab11a were inserted into pcDNA5/FRT/TO-Flag or pcDNA3.1-Myc for mammalian expression. The human Rab11a Q70A mutant was generated by PCR and then inserted into pcDNA5/FRT/TO-EGFP. For the bacterial expression of His-OSBP_321-409_-His, OSBP_321-409_ was inserted into the pET28c vector. For the coexpression of NMT1 and Arf1 (Q71L)-Flag in bacteria, the constructs were inserted into multiple cloning sites 1 and 2, respectively, of the pETDuet-1 vector (Novagen). The pET21a-streptavidin-Alive construct was purchased from Addgene (20860).

### Antibodies

The rabbit polyclonal anti-Rab11a, anti-GFP, and anti-GST antibodies were prepared as previously described (Atik et al., 2014; Sobajima et al., 2014; Kunii et al., 2016). The other primary antibodies included mouse monoclonal Rab11 (clone 47; BD), EEA1 (clone 14; BD), p230 (clone 15; BD), CD-MPR, and Lamp2 (clones 22d4 and H4B4, respectively; Developmental Studies Hybridoma Bank), TfnR (clone H68.4; Zymed), Myc (9B11; Cell Signaling Technology), GAPDH (6C5; EMD Millipore), Flag (M2; Sigma-Aldrich), His (GE Healthcare), sheep TGN46 (Serotec), rabbit calnexin (for immunoblotting, clone C5C9 from Cell Signaling Technology; for immunoisolation, ADI-SPA-860 from Enzo Life Sciences), rat Lamp2 (for mouse, clone Abl-93; Developmental Studies Hybridoma Bank), goat lamin B (C-20; Santa Cruz Biotechnology), rabbit TGN46 (ab50595; Abcam), and rabbit Lamp1 (PA1-654A; Invitrogen). Species-specific secondary antibodies labeled with Alexa Fluor 488, 568, and 594; Cy5; and HRP were purchased from Thermo Fisher Scientific and Jackson ImmunoResearch Laboratories, Inc. The rabbit polyclonal anti-RELCH and anti-OSBP antibodies were raised against bacterially expressed His-RELCH and His-OSBP_321-409_-His, respectively. The antisera were affinity purified and subsequently used for the immunofluorescence and immunoblotting analyses.

### Protein expression and purification

XL-1 blue cells expressing pQE32- or pMAL-based constructs and Rosetta II cells expressing pFAT2- or pET-based constructs were cultured in LB medium at 18°C for 16–18 h with 0.25 mM IPTG and subsequently purified using Ni-NTA agarose (QIAGEN) or amylose resin (New England Biolabs, Inc.) as previously described (Nakajo et al., 2016). The protein-bound Ni-NTA agarose or amylose resin was washed four times with IMAC20 (20 mM Tris-HCl, pH 8.0, 300 mM NaCl, and 20 mM imidazole) or PBS and then eluted with IMAC200 (IMAC20 with 200 mM imidazole) or PBS containing 10 mM maltose, respectively. The proteins were dialyzed in PBS. To purify the nucleotide-free Rab11a (S25N and Q70A)-His, the clarified lysates from bacteria expressing Rab11a S25N and Q70A in pET28c were mixed with cOmplete His-tag purification resin (Roche). After incubating for 2 h at 4°C, the beads were washed twice with IMAC20 and twice with IMAC20 containing 10 mM EDTA, and then the bound proteins were eluted with IMAC200. The eluted proteins were analyzed on SDS-PAGE gels stained with Coomassie brilliant blue (CBB). The peak fractions were dialyzed in PBS. The myristoylated Arf1 (Q71L)-Flag protein was produced and purified as previously described (Franco et al., 1995) with slight modifications. To prepare the myristate–BSA complex, sodium myristate (198-09512; Wako Pure Chemical Industries) was mixed with 5% BSA (A7906; Sigma-Aldrich) in PBS and dissolved by sonication (Branson Sonifier 250) at 60–70°C. Rosetta II cells expressing Arf1 (Q71L)-Flag and NMT1 in pETDuet-1 were grown at 37°C to OD_600_ 0.6–0.7, and prewarmed myristate-BSA was slowly added to a final concentration of 100 µM. After incubating with myristate–BSA for 10 min at 37°C, the bacteria were cultured at 27°C for 18 h with 0.3 mM IPTG. The bacterial pellet was lysed with 1 mg/ml lysozyme (Wako Pure Chemical Industries) in PBS for 30 min at 37°C and sonicated on ice. The lysate was clarified by centrifugation at 12,000 *g* for 5 min. Myristoylated Arf1 was precipitated with 35% ammonium sulfate. The precipitated pellet was dissolved in PBS and incubated with anti-Flag M2 affinity gel (Sigma-Aldrich) for 18 h at 4°C. Then, the beads were washed with NL100 (20 mM Hepes-NaOH, pH 7.5, 100 mM NaCl, 5 mM MgCl_2_, and 0.1% Triton X-100), NL500 (NL100 with 500 mM NaCl), NE200 (NE100 with 200 mM NaCl), and twice with TBS. The protein was eluted with 200 µg/ml Flag-peptide (Sigma-Aldrich) in TBS and analyzed on an SDS-PAGE gel stained with CBB. To prepare the untagged RELCH, XL-1 blue cells expressing pQE32-RELCH containing an N-terminal PreScission protease recognition site were cultured in LB medium at 18°C for 16 h with 0.25 mM IPTG and then purified using Ni-NTA agarose as described above. The bound protein was eluted with IMAC200, and the eluted protein was incubated with 20 U/ml PreScission protease (GE Healthcare) for 3 h at 4°C. Then, the protein was mixed with Ni-NTA beads and Glutathione Sepharose 4B (GE Healthcare) in a dialysis tube and incubated in PBS for 16 h at 4°C to remove the cleaved fragment and the GST-fused PreScission protease. After the beads were removed, the supernatant containing the protein was analyzed by immunoblotting using His and RELCH antibodies to confirm the deletion of the His-tag. To purify Flag-OSBP from mammalian cells, Flp-In–293 cells (Thermo Fisher Scientific) were used to generate a stable Flag-OSBP cell line. The stably transfected cells from fifty 15-cm dishes were harvested and lysed with 0.2% Triton X-100 and 1 mM methyl-β-cyclodextrin (MβCD; Wako Pure Chemical Industries) in PBS. The lysate was clarified and mixed with anti-Flag M2 affinity gel. After incubation for 2 h at 4°C, the beads were washed once with NL100, once with NL500, and twice with PBS. Then, the bound protein was eluted with 400 µg/ml Flag-peptide in PBS. The eluted protein was analyzed on an SDS-PAGE gel stained with CBB. The concentration of the peak fractions was estimated by comparison to a series of BSA standards using SDS-PAGE and CBB staining. Small aliquots of purified proteins were snap-frozen in liquid N_2_ for storage at −80°C.

### Pulldown assay

For the GST pulldown assay using the GST-Rab11a proteins and a mouse brain lysate, 500 µg of the GST-Rab11a (WT or Q70A) protein were bound to 50 µl Glutathione Sepharose 4B in a 4-ml total volume of NE100 (20 mM Hepes-NaOH, pH 7.5, 100 mM NaCl, 20 mM EDTA, and 0.1% Triton X-100) for 1 h at 4°C. The beads were washed three times with NE100 and once with NL100 and incubated for 1 h at 4°C in a 5-ml total volume of 20 mg mouse brain lysate prepared with NL100 containing 10 mM MgCl_2_, 1% protease inhibitor mixture (Wako Pure Chemical Industries), and 10 mM GDP or GTP, pH 7.0 (Wako Pure Chemical Industries). The beads were washed three times with NL100 and once with NL200 (NL100 with 200 mM NaCl), and the bound proteins were subsequently eluted with 500 µl NE200 (NE100 with 200 mM NaCl). The eluted proteins were precipitated with TCA. The proteins were dissolved in 40 µl SDS-PAGE sample buffer, separated on a 4–12% gradient gel, and silver stained. For the GST pulldown assay using the GST-Rab11a and His-RELCH proteins, 10 µg GST-Rab11a WT or Q70A was bound to 20 µl Glutathione Sepharose 4B, and the beads were incubated with 20 µg His-RELCH protein in a 500-µl total volume. For the GST pulldown assay using the recombinant GST-RELCH_497-779_ (Rab-binding domain [RBD]) and Rab11a-His proteins, 10 µg GST-RELCH (RBD) protein were bound to 20 µl Glutathione Sepharose 4B, and the beads were incubated with 10 µg Rab11a (S25N or Q70A)-His in a 500-µl total volume. The eluted protein and bead-bound protein were analyzed by immunoblotting. For the His-tag pulldown assay using the His-RELCH and Flag-OSBP proteins, 0.5 µg His-RELCH protein was bound to 20 µl Ni-NTA beads in a 500-µl total volume of buffer (20 mM Hepes-NaOH, pH 7.5, 100 mM NaCl, and 5 mM MgCl_2_) for 1 h at 4°C. The beads were washed five times with the same buffer and incubated with 0.5 µg Flag-OSBP protein in a 500-µl total volume of the buffer mixed with 10 µl ethanol, 1 mM cholesterol (08722-94; Nacalai Tesque), or 1 mM 25-OH (H1015; Sigma-Aldrich) for 1 h at 4°C. The beads were washed five times with buffer containing ethanol, 20 µM cholesterol, or 20 µM 25-OH and then eluted with 30 µl IMAC200. The eluted proteins were immunoblotted.

### Immunoprecipitation

For the immunoprecipitation assay using the anti-RELCH antibody and a mouse brain lysate, preimmunized rabbit serum or rabbit antiserum against the recombinant RELCH protein was mixed with 40 µl protein G Sepharose 4 Fast Flow (GE Healthcare) in a 150-µl total volume of NE100. After incubation for 1 h at 4°C, the beads were washed five times with 500 µl NE100 and once with NL100 and subsequently incubated for 2 h at 4°C in a 500-µl total volume of 10 mg of a mouse brain lysate prepared with NL100 containing 0.1% protease inhibitor. The beads were washed with NL100, and the bead-bound proteins were dissolved in 40 µl SDS-PAGE sample buffer. For the immunoprecipitation assay using the lysate from the HEK293FT or Flp-In–293 cells coexpressing the indicated proteins, the cells were plated at a density of 5 × 10^5^ cells per well in a six-well plate and transfected with 1 µg of the indicated plasmids. After 24 h, the cells were lysed with 500 µl lysis buffer (NL100 or NL200) containing 0.1% protease inhibitor for 20 min on ice. The lysates were clarified by centrifugation, and the cleared lysates were preabsorbed with 30 µl of Pierce control agarose resin (26150; Thermo Fisher Scientific) for 1 h at 4°C. After removal of beads by centrifugation, the supernatant was incubated with 30 µl anti-Flag M2 affinity gel for 2 h at 4°C. The beads were washed with lysis buffer, and the bound proteins were eluted with 30 µl of 200 µg/ml Flag-peptide in lysis buffer. The eluted proteins were analyzed by immunoblotting.

### Mass spectrometric analysis

The proteins were separated on a NuPAGE Novex Bis-Tris gel (NP0321; Thermo Fisher Scientific) and silver stained using a silver stain mass spectrometry kit (299-58901; Wako Pure Chemical Industries) according to the manufacturer’s instructions. The bands were cut from the gel, and the proteins were reduced, alkylated, and digested with trypsin in Tris buffer for 16 h at 37°C. The samples were analyzed using a SYNAPT G2 (Waters Corp.) or QExactive (Thermo Fisher Scientific) mass spectrometer at Osaka University Center for Medical Research and Education. The database search was conducted using ProteinLynx Global Server (v.2.4; Waters Corp.) or MASCOT Server (v.2.3; Matrix Science) software and the International Protein Index database (mouse; v.3.77 or 3.87; EMBL-EBI).

### Yeast two-hybrid assay

The yeast two-hybrid assay was performed as previously described (Haas et al., 2005). The *Saccharomyces cerevisiae* strain PJ69-4A was cotransformed with Gal4BD (pFBT9-Rabs) and Gal4AD (pACT2) plasmids harboring full-length or deletion constructs of RELCH and grown on SC-LW (SC/−Leu/−Trp) plates for 3 d at 30°C; five independent colonies were selected and restreaked on SC-LW and QDO (SC/−Leu/−Trp/−His/−Ade) plates, and the cells were grown for 3 d at 30°C.

### Cell culture and transfection

HeLa, HEK293FT, and Flp-In–293 cells were cultured in DMEM with high glucose, l-glutamine, phenol red, and sodium pyruvate (Wako Pure Chemical Industries) containing 10% FBS at 37°C and 5% CO_2_. Flp-In–293 cells stably expressing Flag-OSBP and Flag-RELCH were maintained in medium containing 100 µg/ml hygromycin B. For the plasmid transfection, TransIT-LT1 transfection reagent (Mirus Bio) was used as previously described (Nakajo et al., 2016). For the siRNA transfection, Lipofectamine RNAiMAX transfection reagent (Thermo Fisher Scientific) was used according to the manufacturer’s instructions. The siRNA oligonucleotides used in this study included Hs_RAB11A_5 (SI00301553; QIAGEN), Hs_RAB11B_6 (SI02662695; QIAGEN), Hs_OSBP_5 (SI02628920; QIAGEN), and Hs_KIAA1468_4116_s and _as (11861007 and 11861008; Sigma-Aldrich). The RNAi depletion efficiencies were confirmed by immunoblotting.

### Generation of shRNA lentivirus and stable cell lines

shRNA lentiviruses expressing Rab11a, RELCH, and OSBP shRNA were produced using a pLKO.1 vector, and the lentivirus expressing Rab11b-shRNA was produced using a pLKO.1-neo vector (10878 and 13425; Addgene) according to the manual. The following oligonucleotides were designed: 5′-CCGGAAGAGTAATCTCCTGTCTCGACTCGAGTCGAGACAGGAGATTACTCTTTTTTTG-3′ (Hs_Rab11a_shRNA_F) and 5′-AATTCAAAAAAAGAGTAATCTCCTGTCTCGACTCGAGTCGAGACAGGAGATTACTCTT-3′ (Hs_Rab11a_shRNA_R); 5′-CCGGCCGCATCGTGTCACAGAAACACTCGAGTGTTTCTGTGACACGATGCGGTTTTTG-3′ (Hs_Rab11b_shRNA_F) and 5′-AATTCAAAAACCGCATCGTGTCACAGAAACACTCGAGTGTTTCTGTGACACGATGCGG-3′ (Hs_Rab11b_shRNA_R); 5′-CCGGCCAATCAAACCTCTTGAAACTCGAGTTTCAAGAGGTTTGATTGGTTTTTG-3′ (Hs_RELCH_shRNA_F) and 5′-AATTCAAAAACCAATCAAACCTCTTGAAACTCGAGTTTCAAGAGGTTTGATTGG-3′ (Hs_RELCH_shRNA_R); and 5′-CCGGCCCGCTAATGGAAGAAGTTTACTCGAGTAAACTTCTTCCATTAGCGGGTTTTTG-3′ (Hs_OSBP_shRNA_F) and 5′-AATTCAAAAACCCGCTAATGGAAGAAGTTTACTCGAGTAAACTTCTTCCATTAGCGGG-3′ (Hs_OSBP_shRNA_R). HeLa cells were infected with the produced viruses as previously described (Nakajo et al., 2016). The cells were cultured with growth medium (DMEM plus 10% FBS) containing 2 µg/ml puromycin for RELCH or OSBP shRNA or medium containing 2 µg/ml puromycin and 400 µg/ml G418 for Rab11a/b shRNA for 7 d. The cells were seeded at a density of a single cell per well in 96-well plates to obtain single clones. The depletion efficiency of each clone was examined by performing immunofluorescence and immunoblotting analyses.

### Immunofluorescence and transport assay

HeLa cells were seeded on glass coverslips. The cells were washed with PBS, fixed with 3% PFA in PBS for 20 min at RT, and washed again with PBS. The fixed cells were permeabilized with 0.2% saponin in PBS for 4 min at RT and incubated with the primary antibodies in PBS containing 0.2% saponin for 1 h at RT. The cells were washed with PBS and incubated with the secondary antibodies and/or DAPI in PBS containing 0.2% saponin for 1 h at RT. After washing with PBS, the coverslips were mounted in Mowiol mounting medium (0.1 M Tris-HCl, pH 9.5, 25% glycerol, 10% Mowiol, and 5% DABCO). The VSVG transport assay and surface biotinylation assay were performed as previously described (Diao et al., 2008). 5 × 10^5^ HeLa cells were incubated with the growth medium containing the adenovirus encoding VSVG^tsO45^-GFP for 1 h at 37°C and then further cultured for 24 h at 40°C. VSVG^tsO45^-GFP was released from the ER at 32°C in the presence of 0.1 mg/ml cycloheximide (C1988; Sigma-Aldrich). At the indicated times, the cells were washed twice with ice-cold PBS and incubated with 500 µl PBS supplemented with 5 mg/ml sulfo-NHS-LC-biotin (21327; Thermo Fisher Scientific) for 30 min at 4°C. The cells were washed twice with ice-cold PBS and quenched by 50 mM NH_4_Cl in PBS for 15 min at 4°C. After washing again, the cells were lysed, and the cleared lysate was incubated with 20 µl streptavidin beads (N-1000; SoluLink) for 1 h at 4°C. The beads were washed three times in ice-cold PBS with 0.5% Triton X-100, and the bound proteins were analyzed by immunoblot using anti-GFP antibody. The transferrin uptake assay was performed as previously described (Linford et al., 2012). 5 × 10^4^ HeLa cells seeded on coverslips were incubated with serum-free DMEM for 16 h. The cells were then incubated with uptake medium (DMEM with 20 mM Hepes-NaOH, pH 7.5, and 2% BSA) containing 5 µg/ml Alexa Fluor 594–conjugated transferrin (Molecular Probes) for 1 h at 4°C. After washing with ice-cold PBS, the transferrin-bound cells were incubated with growth medium at 37°C and fixed at the indicated times. The fixed cells were analyzed by immunofluorescence using primary (anti-TfnR) and secondary (Alexa Fluor 488–labeled anti–mouse) antibodies. For TGN38 and Furin endocytosis assays, HeLa cells on glass coverslips were transfected with pcDNA3.1 plasmid encoding Flag-TGN38 (Shin et al., 2004) and Furin (Chia et al., 2011) for 24 h. After washing with ice-cold PBS, the coverslips were incubated with 50 µl uptake medium with 0.5 µg/ml anti-Flag M2 antibody dropped on an ice-cold metal plate for 45 min. The coverslips were washed with ice-cold PBS, and then one coverslip was fixed with 3% PFA (*t* = 0 min). The other coverslips were further incubated in prewarmed growth medium at 37°C and fixed at the indicated time points. The fixed cells were analyzed by immunofluorescence using primary (anti-TGN46) and secondary (Alexa Fluor 488–labeled anti–sheep and Alexa Fluor 568–labeled anti–mouse) antibodies. For 25-OH treatment, the cells were incubated with 6.2 µM 25-OH or solvent (ethanol) in growth medium for 24 h before analysis. For Filipin staining of the HeLa cells, the cells on glass coverslips were fixed and stained as described above. After staining with the primary and secondary antibodies, the cells were washed with PBS and stained with 50 µg/ml Filipin (Sigma-Aldrich) in PBS for 2 h at RT. Then, the coverslips were mounted. For rhodamine-dextran uptake analysis, the cells were incubated with growth medium containing 1 mg/ml rhodamine-dextran (D1824; Molecular Probes) for 3 h before fixing and staining with Filipin and/or Lamp2 as described above. For BODIPY-cholesterol staining of the HeLa cells, the cells were incubated with growth medium containing 1 µg/ml BODIPY-cholesterol (810255; Avanti Polar Lipids) or solvent (DMSO) for 24 h before fixation. For U18666A treatment, the cells were incubated with 2 µg/ml U18666A (U3633; Sigma-Aldrich) or solvent (ethanol) in growth medium for 16 h before analysis. The fixed cells were imaged under an FV1000D confocal microscope with an UPlan S-Apochromat 100× 1.4 NA oil-immersion objective using FluoView software (Olympus). For SR-SIM, the samples were imaged under an ELYRA S.1 microscope with a 100× 1.46 NA oil immersion objective (ZEISS). The images and fluorescence intensity profiles were analyzed using ZEN 2011 software (ZEISS).

### Immunoelectron microscopy

HeLa cells cultured in 35-mm plastic dishes were transfected with EGFP-Rab11a. 4 h after transfection, the cells were treated with 6.2 µM 25-OH overnight then fixed with 2% PFA and 0.1% glutaraldehyde in PBS, pH 7.4, for 30 min at RT; washed with PBS; and permeabilized in 5% normal goat serum and 0.25% saponin in PBS. After quenching with 1 mg/ml NaBH_4_ in the same solution for 30 min at RT, the cells were washed with PBS and incubated with rabbit antisera against GFP (1:100; Atik et al., 2014) for 1 h at 37°C and then with Alexa Fluor 594–labeled FluoroNanogold (1:10; Nanoprobes) in 5% normal donkey serum and 0.25% saponin in PBS for 1 h at RT. The cells were further washed with PBS and postfixed in 1% glutaraldehyde in PBS for 15 min. The fixed cells were incubated with HQ silver enhancement solution (Nanoprobes) for 10 min at RT, extensively washed with distilled water, and incubated with selenium toner (1:20; Kodak) for 7 min at RT to prevent erosion of the silver during the OsO_4_ fixation. After washing with distilled water, the cells were fixed with 2% glutaraldehyde in Hepes buffer (30 mM Hepes, 100 mM NaCl, and 2 mM CaCl_2_, pH 7.4) for 10 min, washed with 10% sucrose, and postfixed in 1% osmium tetroxide in the same buffer for 1 h on ice. The fixed cells were washed with distilled water, stained with 0.5% uranyl acetate in distilled water overnight, dehydrated, and embedded as previously described (Kunii et al., 2016).

### Generation of RELCH KO mice using the CRISPR-Cas9 system

RELCH KO mice (C57BL6/J mouse background) were generated as previously described (Nakajo et al., 2016). We used guide RNA (5′-GATGGAGGCGCCAGGGATCCC-3′) targeting the first coding exon of the RELCH locus. The mutation of the RELCH gene was confirmed by DNA sequencing. The absence of the RELCH protein was confirmed by immunoblotting analysis. Mouse tail–tip fibroblasts were isolated according to a standard protocol. All animal procedures were performed according to the guidelines of the Animal Care and Experimentation Committee of Osaka University, and all animals were bred at the Institute of Animal Experimental Research of Osaka University.

### Subcellular fractionation, immunoisolation of membranes, and cholesterol measurement

Subcellular fractionation was performed as previously described (Yoshimura et al., 2001) with slight modifications as follows. HeLa cells (4 × 10^7^) stably expressing the indicated shRNA were washed once with 20 ml PBS. The cells were scraped in PBS and centrifuged at 1,000 *g* for 5 min. The cell pellets were suspended in 500 µl homogenization buffer (HB; 25 mM Tris-HCl, pH 7.3, 130 mM KCl, and 5 mM MgCl_2_) containing a protease inhibitor and homogenized by passing through a 27-gauge needle 20 times. The homogenate was centrifuged twice at 1,000 *g* for 5 min to collect the postnuclear supernatant (PNS). The PNS was layered on top of a step density gradient (3 ml of 5%, 2 ml of 10%, 2 ml of 12.5%, 1 ml of 15%, 1 ml of 20%, 2 ml of 25%, and 0.5 ml of 40% Histodenz [D2158; Sigma-Aldrich] in HB) in a 13.2-ml Ultra-Clear centrifuge tube (Beckman Coulter) and centrifuged at 100,000 *g* using an SW41Ti rotor (Beckman Coulter) for 4 h at 4°C. The 12 fractions were collected from top to bottom and snap-frozen in liquid nitrogen for storage at −30°C. For immunoisolation of the LE/lysosome, TGN, and ER membranes, 200 µg PNS was preincubated with Dynabeads protein G (Invitrogen) precoated with 1 mg/ml BSA for 1 h, and the PNS was incubated with 1 µg Lamp1, TGN38, and calnexin antibodies, which recognize the cytoplasmic region of each protein. The beads were washed three times with HB. Each fraction was analyzed by immunoblotting, and the cholesterol content was measured using an Amplex red cholesterol assay kit (Molecular Probes) according to the manufacturer’s instructions. After incubating at 37°C for 30 min in a 96-well plate (260836; Nunc), the fluorescence intensity was measured at an excitation wavelength of 545 nm and an emission wavelength of 590 nm using a microplate reader (SH-9000 Lab; Corona Electric). The cholesterol content was calculated according to a cholesterol standard curve and normalized to the protein content. The data are presented as the percentage of total cholesterol (µg/mg protein).

### Liposome preparation

The liposomes were prepared as previously described (Mesmin et al., 2013). The following lipids were used in this study: egg PC, brain PS, liver PI, brain PI4P, liver PE, dansyl-PE, rhodamine-PE (Rho-PE), DOGS-NTA (Ni), and biotin-PC (840051, 840032, 840042, 840045, 840026, 810330, 810150, 790404, and 860563, respectively; Avanti Polar Lipids); OG-PE (O12650; Molecular Probes); and cholesterol and DHE (E2634; Sigma-Aldrich). The cholesterol and DHE stock solutions were maintained in methanol, and the other lipids were maintained in chloroform. The lipid stock solutions in chloroform or methanol were mixed as described below, and the solvent was evaporated using a centrifugal concentrator (EZ-2 Plus; Genevac). The dried lipid films were hydrated at 50°C for 10 min under regular vortex in 500 µl of 1× HKM buffer (50 mM Hepes-NaOH, pH 7.2, 120 mM potassium acetate, and 5 mM MgCl_2_), giving a total lipid concentration of 1–5 mM. The suspensions were frozen and thawed five times using liquid N_2_ and a water bath set at 42°C and extruded 20 times through polycarbonate filters with a pore size of 0.1 µm (Avanti Polar Lipids) using a Mini-Extruder (Avanti Polar Lipids) according to the manufacturer’s instructions. The extruded liposomes were stored in the dark at 4°C and used within 2 d. The following standard molar ratios of liposomes were used: Golgi-like (acceptor) liposomes PC/PS/PI/PI4P/PE = 63/5/10/2/20 mol% and RE-like (donor) liposomes PC/PS/DOGS-NTA (Ni)/cholesterol or DHE = 74/4/4/18 mol%.

### Liposome flotation and sedimentation assay

The liposome flotation assay was performed as previously described (Xiong et al., 1999; Bigay et al., 2005) with slight modifications. To prepare the BSA-coated tubes, polycarbonate tubes (343778; Beckman Coulter) were incubated with 0.1 mg/ml BSA (23209; Thermo Fisher Scientific) in PBS at RT for 2 h, the solution was removed, and the tubes were dried overnight at RT. The myristoylated Arf1 (Q71L)-Flag (400 nM) and Flag-OSBP (600 nM) proteins were incubated with the Golgi-like liposomes (100 µM) in a 50-µl total volume of 1× HKM buffer with 5 mM GTP at 4°C for 30 min and mixed with 150 µl of 75.6% sucrose in 1× HKM buffer, which was prepared using 10× HKM buffer and 84% sucrose in deionized water. The mixture was transferred to a BSA-coated polycarbonate tube and overlaid with 200 µl of 41.2% sucrose and 100 µl of 17.2% sucrose in 1× HKM buffer. The sample was centrifuged at 350,000 *g* using a TLA120.2 rotor (Beckman Coulter) for 2 h at 4°C. The top and bottom fractions (250 µl each) were collected. Each fraction was analyzed by SDS-PAGE and immunoblotting. For the liposome sedimentation assay, the Rab11a (Q70A)-His protein (5 µM) was incubated with the RE-like liposomes (200 µM) in a 30-µl total volume of 1× HKM buffer with 5 mM GTP at 4°C for 30 min and then untagged RELCH (50 nM) in 30 µl of 1× HKM buffer at 4°C for 30 min. The mixture was transferred to a BSA-coated polycarbonate tube and mixed with 440 µl of 1× HKM buffer with 1 mM GTP. The sample was centrifuged at 100,000 *g* using a TLA120.2 rotor for 30 min at 4°C. The supernatant and pellet were analyzed by SDS-PAGE and immunoblotting.

### Silica bead–based liposome binding assay and liposome tethering assay

For the experiments using the silica bead–based liposome, the Golgi-like liposomes were supplemented with 2 mol% Rho-PE and/or 2 mol% biotin-PC, and the RE-like liposomes were supplemented with 2 mol% Rho-PE or OG-PE. The liposome-coated silica beads were prepared as previously described (Mesmin et al., 2013). Liposomes containing Rho-PE or OG-PE (20–50 µM total lipids) were incubated with 5 × 10^6^ silica beads with a size of 5 µm (SS05N; Avanti Polar Lipids) in a 100-µl total volume of 1× HKM buffer at RT for 30 min under regular vortexing. The beads were washed with 1× HKM buffer and suspended in 50 µl of 1× HKM buffer. For the liposome binding assay of the Golgi-like liposomes containing Rho-PE, 5 µl of the suspension (1 µM total lipids) was incubated with or without the myristoylated Arf1 (Q71L)-Flag (200 nM) protein for 30 min at 4°C and then incubated with or without Flag-OSBP (40 nM) for 30 min at 4°C in 100 µl of 1× HKM buffer containing 1 mM GTP and 0.02 mg/ml BSA (1× HKMGB). The beads were washed with 1× HKMGB and incubated with the primary antibodies (Flag or OSBP antibodies) in 100 µl of 1× HKMGB for 1 h at RT. The beads were washed and incubated with Alexa Fluor 488–labeled secondary antibodies at RT. After washing, the beads were gently suspended in 20–30 µl of 1× HKM buffer and mounted on a glass slide. For the liposome binding assay of RE-like liposomes containing Rho-PE, 5 µl of the suspension (2.5 µM total lipids) was incubated with or without 5 µM Rab11a (Q70A)-His for 30 min at 4°C and then incubated with or without untagged RELCH (200 nM) for 30 min at 4°C in 100 µl of 1× HKMGB. The beads were stained with primary antibodies (Rab11 or RELCH) and Alexa Fluor 488–labeled secondary antibodies as described above. For the liposome tethering assay, Arf1/OSBP (200/160 nM) and Rab11a-His/untagged RELCH (5 µM/75 nM) were bound to beads coated with Golgi-like liposomes containing Rho-PE and RE-like liposomes containing OG-PE, respectively, as described above. The protein-bound beads were mixed in a 100-µl total volume and incubated for 30 min at 4°C. After removing the supernatant, the beads were gently suspended in 20 µl of 1× HKM buffer, mounted on glass slides, and immediately imaged under a BX61 microscope with UPlan Apochromat 20× 0.75 NA or UPlan Apochromat 40× 0.85 NA objectives and cellSens software (Olympus). For the tethering assay using streptavidin-His protein and biotin-PC, streptavidin-His (1 µM) was bound to beads coated with RE-like liposomes containing OG-PE, and the protein-bound beads were mixed with beads coated with Golgi-like liposomes containing Rho-PE and biotin-PC. The liposome tethering efficiency was quantified as follows: the total numbers of beads and contacts between the rhodamine-labeled beads and Oregon green 488–labeled beads were counted in at least 40 randomly selected fields per group. Statistical analysis was conducted using JMP Pro 11.0 software (SAS Institute).

### Cholesterol transfer assay

For the cholesterol transfer assay, Golgi-like (Acceptor) liposomes supplemented with 2.5 mol% dansyl-PE and/or 2 mol% biotin-PC and RE-like (donor) liposomes containing DHE were used as acceptor and donor liposomes, respectively. Lipid transfer reactions between donor and acceptor liposomes were performed in a 100-µl volume. The final concentration of the total lipids in the reaction was 0.35–1 mM, and the donor and acceptor liposomes were added at a 1:6 ratio. The protein-bound liposomes were incubated with Rab11a-His (3.5 µM) and Arf1 (100 nM) for 15 min at 4°C and then incubated with untagged RELCH and OSBP (200 nM), respectively, in 100 µl of 1× HKM buffer containing 5 mM GTP for 15 min at 4°C. For the transfer assay using streptavidin-His and biotin-PC, the donor liposomes were incubated with streptavidin-His (1 µM). The transfer reaction was initiated by mixing the protein-bound donor and acceptor liposomes in a 96-well plate (655076; Greiner Bio-One). FRET between DHE and dansyl, indicating DHE transfer, was measured at an excitation wavelength of 310 nm and an emission wavelength of 525 nm every 1 min over 30 min at RT using a microplate reader (SH-9000 Lab; Corona Electric). As a positive control, MβCD was added to the reaction mixture at a final concentration of 1 mM to determine the maximum FRET signal. Maltose binding protein was used as a control protein. The data from each experiment were corrected by setting the initial and maximum values to 0 and 1, respectively. The results represent at least three independent experiments.

### Quantification and statistical analysis

Data analysis and graph presentation were performed using Excel (Microsoft), ImageJ (National Institutes of Health), JMP Pro 11.0 (SAS Institute), and ZEN 2011 and are reported in the corresponding figure legends and methods. The statistical analyses were performed using Excel and JMP Pro11.0. The data shown in Figs. 3 (D and G), 4 D, 6 B, 7 (B, E, and G), and S3 E were analyzed by performing two-tailed Student’s *t* tests, and the data shown in Fig. 9 A were analyzed by performing ANOVA and Wilcoxon and Kruskal–Wallis tests.

### Online supplemental material

Fig. S1 shows the yeast two-hybrid assay, which was performed to determine the RELCH region involved in the binding to Rab11 and the analysis of the subcellular localization of RELCH. Fig. S2 shows the cargo protein transport and immunoblotting analyses in the knockdown cells. Fig. S3 shows the localization of RELCH and Rab11 after 25-OH treatment and the translocation of RELCH and OSBP to the TGN area in the Rab11-depleted cells. Fig. S4 confirms the cholesterol distribution in RNAi-treated HeLa cells. Fig. S5 shows the liposome binding assay and silica bead–based liposome tethering assay.

## Supplementary Material

Supplemental Materials (PDF)
