# Supplementary material for: The Rab11-binding protein RELCH/KIAA1468 controls intracellular cholesterol distribution
Source: J Cell Biol. 2018 May 7;217(5):1777–96. doi: 10.1083/jcb.201709123 (PMC5940305; doi:10.1083/jcb.201709123)
Supplement: Supplemental Materials (PDF) [file JCB_201709123_sm.pdf]

Supplemental material

Sobajima et al., https://doi.org/10.1083/jcb.201709123

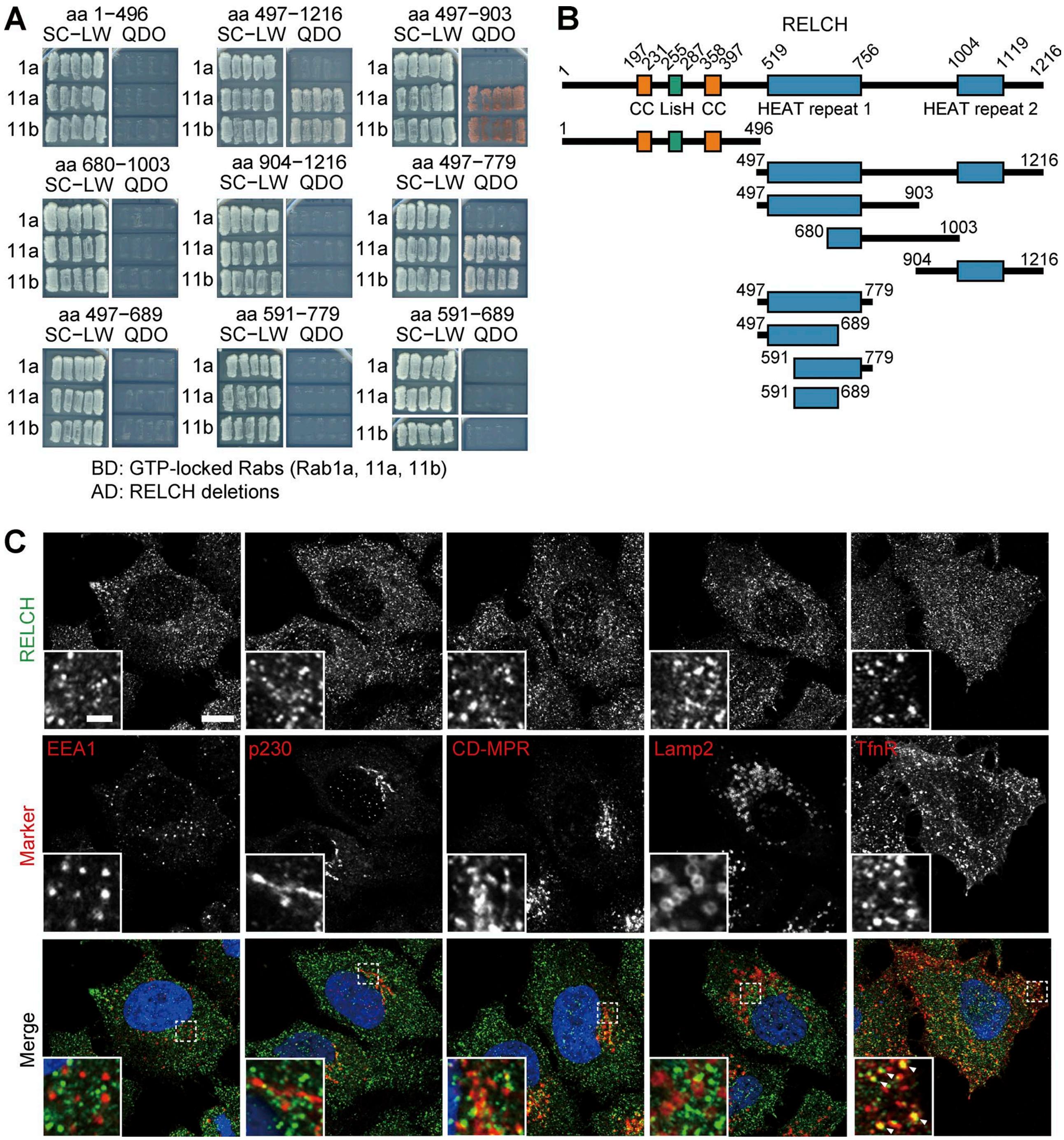

Figure S1. **Determination of the RELCH region that binds Rab11 and analysis of the subcellular localization of RELCH. (A)** The yeast strain cotransformed with Gal4AD (AD) plasmids encoding RELCH deletion mutants and Gal4BD (BD) plasmids encoding GTP-locked Rab1a, Rab11a, or Rab11b was grown on a QDO plate to demonstrate the interaction between the two proteins. **(B)** The deletion constructs used in A are presented. **(C)** HeLa cells were immunostained with antibodies against RELCH and several organelle markers (EEA1, early endosome; p230, TGN and CD-MPR; Lamp2, LE/lysosome). The nuclei were stained with DAPI (blue). The arrowheads indicate that RELCH colocalized with the TfnR-positive compartment. Bars: (full images) 10 µm; (insets) 2 µm.

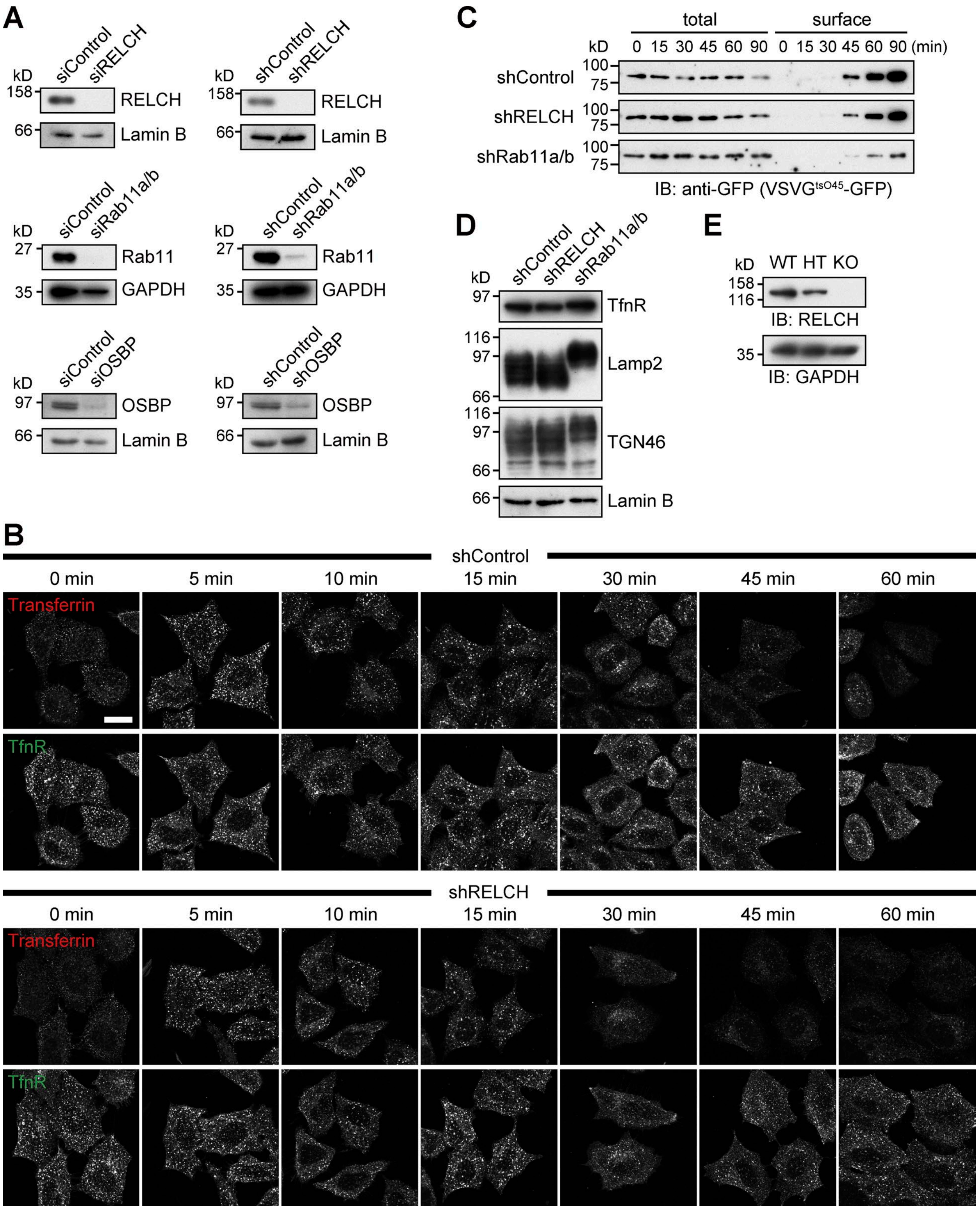

Figure S2. **Cargo protein transport and immunoblotting analyses in knockdown cells. (A)** The siRNA or lentivirus-based shRNA depletion efficiencies of RELCH, Rab11a/b, and OSBP were examined by immunoblotting (IB). **(B)** Transferrin uptake assay in RELCH-depleted cells. The cells were cultured in serum-free medium for 16 h before binding with Alexa Fluor 594–labeled transferrin. The cells were fixed at the indicated time points after incubation at 37°C and immunostained with the TfnR antibody. Bar, 10 µm. **(C)** VSVG transport assay in shRNA-treated HeLa cells. The cells were infected with adenovirus encoding VSVG^{tsO45}-GFP and incubated at 40°C for 24 h, followed by incubation at 32°C to allow the cargo to exit from the ER. At the indicated time points, the cell surface proteins were biotinylated and precipitated using streptavidin beads. Total (input) and surface (bead-bound) VSVG proteins were immunoblotted with the GFP antibody. **(D)** The lysates from the RELCH- or Rab11a/b-depleted HeLa cells were immunoblotted using antibodies against various transmembrane cargo proteins (TfnR, Lamp2, and TGN46) that exit from the TGN. **(E)** Lysates from the brains of WT and RELCH heterozygous (HT) and homozygous KO mice were immunoblotted with the antibody against RELCH. Lamin B and GAPDH were used as loading controls.

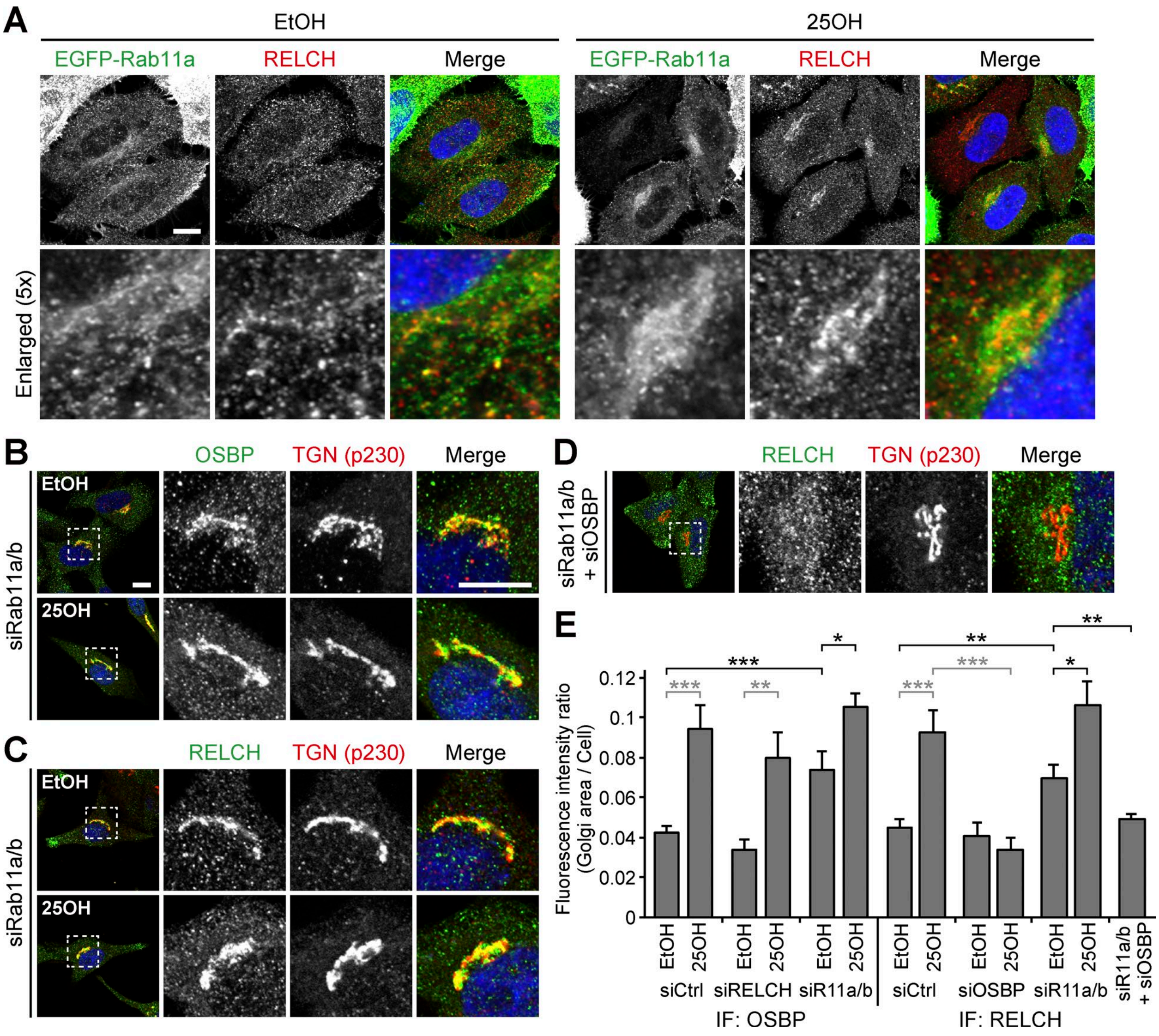

Figure S3. **Localization of RELCH and Rab11 after 25-OH treatment and translocation of RELCH and OSBP to the TGN area in the Rab11-depleted cells. (A)** HeLa cells transfected with EGFP-Rab11a were incubated with solvent (ethanol; EtOH) or 25-OH for 24 h before analysis. The cells were immunostained with the RELCH antibody. **(B–E)** siRNA-transfected HeLa cells were immunostained with antibodies against OSBP (B) and RELCH (C and D) after 25-OH treatment. The TGN was labeled with the p230 antibody. The enlarged images show the TGN area. **(E)** Quantification of the ratio of the fluorescence intensity in the Golgi area to the intensity in the whole intracellular area (n = 10–30 cells). Data are expressed as means ± SEM. Significance was calculated by performing two-tailed Student's t tests (*, $P < 0.05$; **, $P < 0.01$; ***, $P < 0.001$). The nuclei were stained with DAPI (blue). Bars, 10 µm. IF, immunofluorescence.

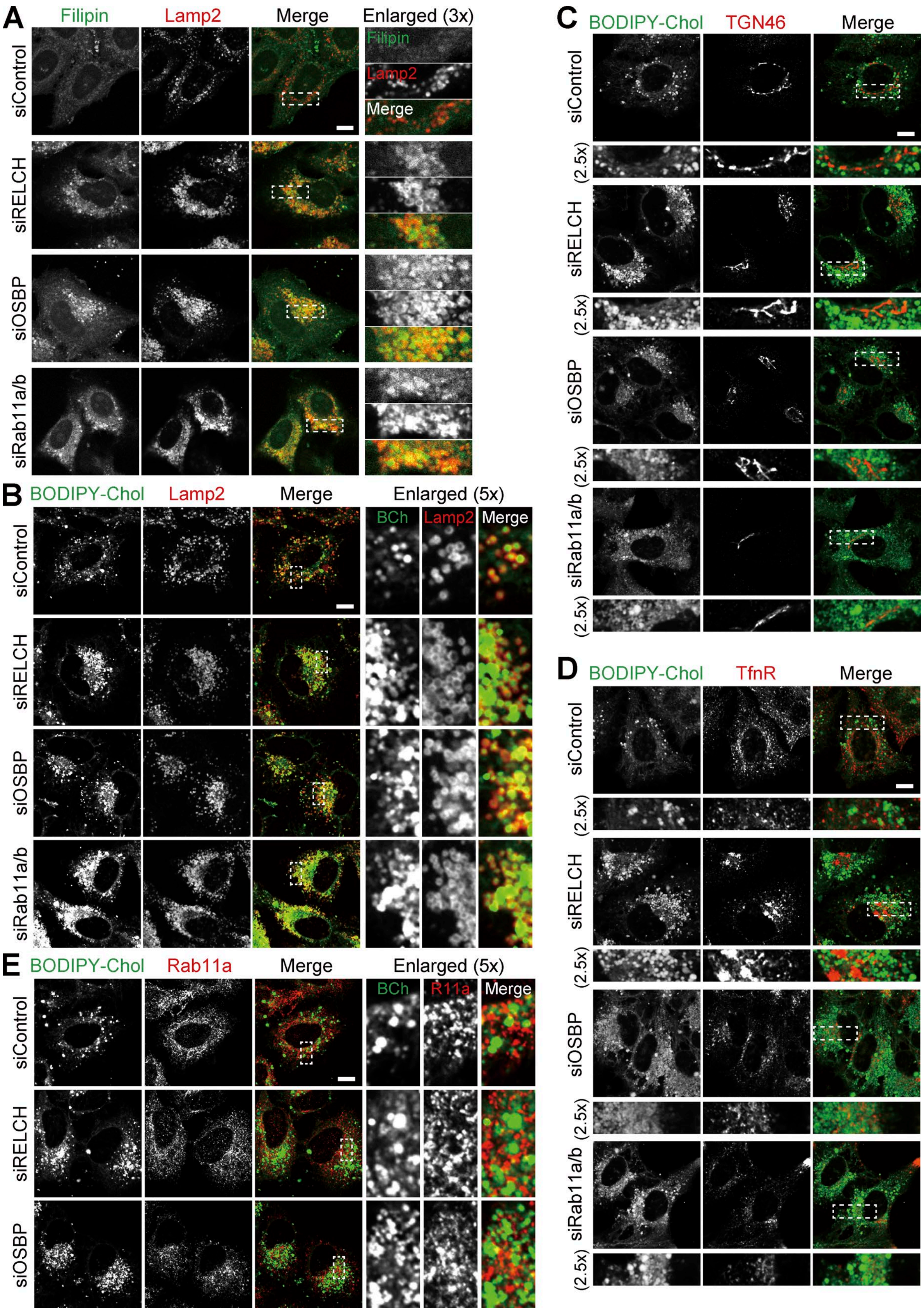

Figure S4. **Cholesterol distribution in the RNAi-treated HeLa cells. (A–E)** HeLa cells transfected with siRNA against RELCH, OSBP, or Rab11 were labeled with Filipin (A) or BODIPY-cholesterol (B–E). The cells were costained with antibodies against Lamp2 (A and B), TGN46 (C), TfnR (D), and Rab11a (E). Bars, 10 µm.

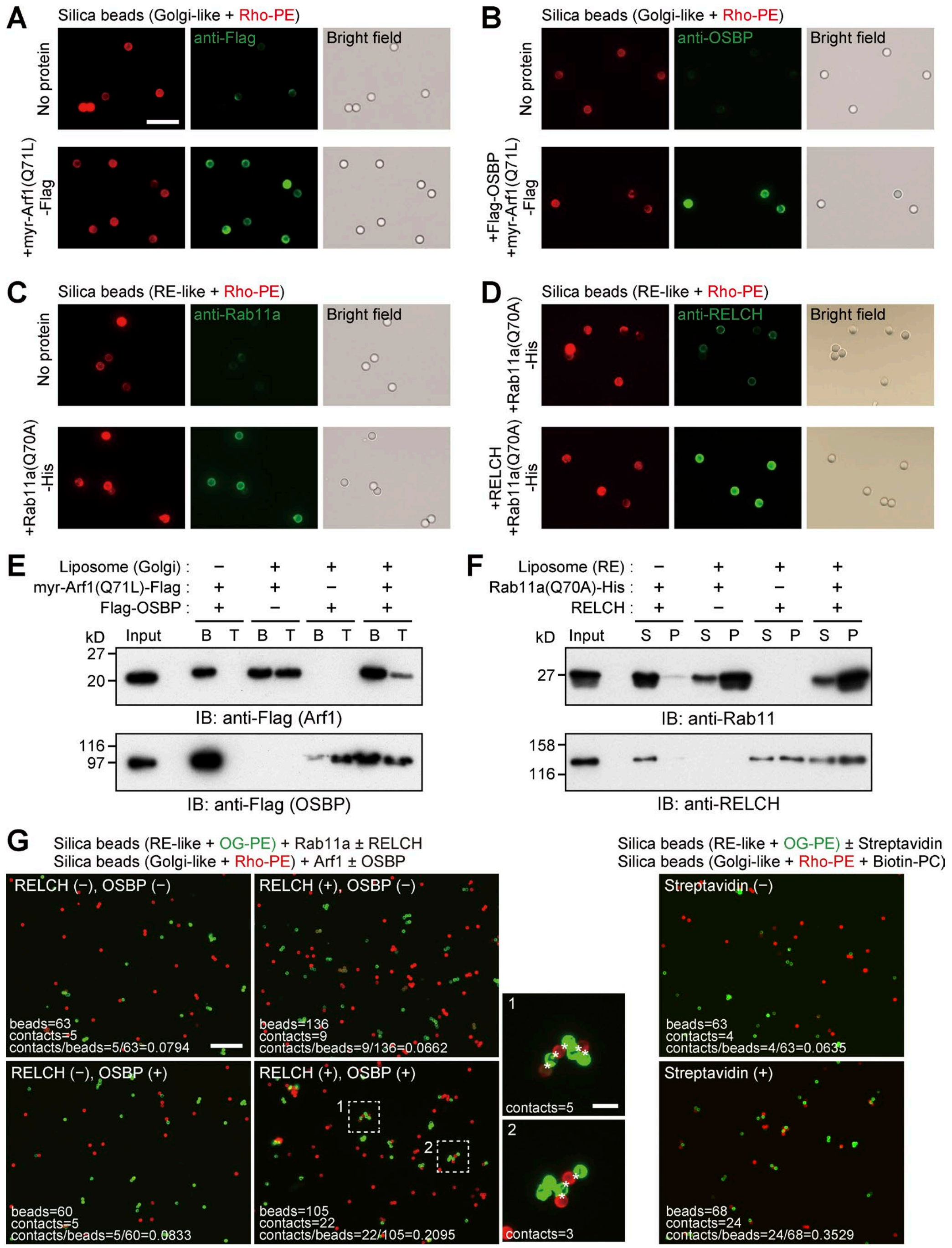

Figure S5. **Liposome binding assay and silica bead–based liposome tethering assay. (A–D)** Silica beads coated with Golgi-like liposomes containing Rho-PE (red) were incubated with the C-terminal Flag-tagged myristoylated Arf1 Q71L mutant (A and B). Beads coated with RE-like liposomes containing Rho-PE (red) were incubated with the C-terminal His6×-tagged Rab11a Q70A mutant (C and D). Golgi-like and RE-like liposomes were further incubated with Flag-tagged OSBP (B) and untagged RELCH (D), respectively. The proteins on the liposomes were immunostained with the indicated antibodies (green). Bar, 20 µm. **(E)** Liposome flotation assay using Golgi-like liposomes, Flag-OSBP, and myr-Arf1 (Q71L)-Flag. These proteins were mixed with or without Golgi-like liposomes in the presence of GTP, and the mixture was adjusted to 57% sucrose and overlaid with two cushions of sucrose at a lower density (41% and 17%). After ultracentrifugation, the top (T) and bottom (B) fractions were collected and analyzed by immunoblotting (IB) using the Flag antibody. **(F)** Liposome sedimentation assay using RE-like liposomes, RELCH, and Rab11a (Q70A)-His. These proteins were incubated with or without RE-like liposomes in the presence of GTP, and the mixture was ultracentrifuged. The supernatant (S) and pellet (P) were immunoblotted using the RELCH and Rab11 antibodies. **(G)** Representative images of the silica bead–based liposome tethering experiment. In the left panel, Golgi-like liposome (containing Rho-PE)-bound myr-Arf1 (Q71L)-Flag and RE-like liposome (containing OG-PE)-bound Rab11a (Q70A)-His were incubated with or without Flag-OSBP and RELCH proteins, respectively. The protein-bound beads were mixed and incubated. After washing, the beads were suspended, mounted on glass slides, and imaged under a fluorescence microscope. In the right panel, the tethering between the streptavidin-bound RE-like liposomes and Golgi-like liposomes containing biotin-PC was assessed. The asterisks in the enlarged fields indicate the contacts between two liposomes. Examples of the calculations of the liposome tethering efficiency are shown. Bars: (main images) 50 µm; (enlarged fields) 10 µm.
